# Utilising Human Myometrial and Uterine Fibroid Stem Cell‐Derived Three Dimentional Organoids as a Robust Model System for Understanding the Pathophysiology of Uterine Fibroids

**DOI:** 10.1111/cpr.70025

**Published:** 2025-03-20

**Authors:** Mervat M. Omran, Somayeh Vafaei, Samar Alkhrait, Farzana Liakath Ali, Maria Victoria Bariani, Tao Bai, Winston E. Thompson, Qiwei Yang, Mohamed Ali, Ayman Al‐Hendy

**Affiliations:** ^1^ Department of Obstetrics and Gynecology University of Chicago Chicago Illinois USA; ^2^ Cancer Biology Department National Cancer Institute ‐ Cairo University Cairo Egypt; ^3^ Department of Family Medicine Johnston Memorial Hospital, Ballad Health Virginia USA; ^4^ Obstetrics and Gynecology, Feinberg School of Medicine Northwestern University Chicago Illinois USA; ^5^ Department of Physiology Morehouse School of Medicine Atlanta Georgia USA; ^6^ Department of Obstetrics and Gynecology Morehouse School of Medicine Atlanta Georgia USA; ^7^ Clinical Pharmacy Department, Faculty of Pharmacy Ain Shams University Cairo Egypt; ^8^ Department of Medical Sciences Khalifa University Abu Dhabi UAE

**Keywords:** 3D organoids, extracellular matrix, racial disparities, uterine fibroid

## Abstract

Uterine fibroids (UFs) are the most common benign gynecologic tumours affecting women of reproductive age. This study aims to deepen the understanding of UFs complex aetiology through harnessing the power of 3D organoid models derived from human myometrial stem cells to emulate the in vivo behaviour of these tumours. Isolated SCs were cultured over 7 days under a defined culture system. Immunohistochemistry, Immunofluorescence, organoid stiffness, RNA Sequencing was conducted, and differential gene expression was assessed using RT‐PCR. The derived organoids exhibited diverse populations of cells, including stem cells, smooth muscle, and fibroblasts. Excessive ECM deposition was shown via Collagen and Fibronectin expression. We confirmed that our organoids expressed oestrogen receptor in a pattern similar to that in their corresponding tissue, as well as responded to steroid hormone. Interestingly, we revealed significant racial disparities in ECM accumulation within organoids derived from different racial groups. This augmented ECM deposition is theorised to enhance tissue stiffness, as assessed using Young's modulus. Additionally, our research demonstrated significant decreases in fibrotic markers upon treatment with Vitamin D3 and Doxercalciferol. Furthermore, the pro‐fibroid effects of environmental phthalates further elucidate the potential factors contributing to UF pathology. The 3D organoid model can serve as a robust platform to study the underlying molecular mechanisms of UFs, besides offering invaluable insights for potential therapeutic interventions.

## Graphical abstract

Three‐dimensional stem cell‐derived organoid model can serve as a robust platform to study the underlying molecular mechanisms of UFs, UFs health disparity, environmental risk factors, besides offering invaluable insights for potential therapeutic interventions.

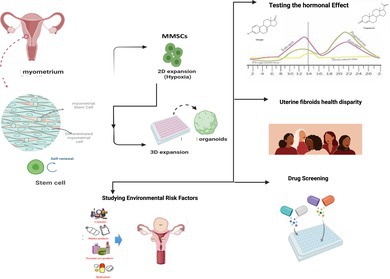

## 1 Background

Uterine fibroids (UFs), also known as Uterine leiomyomata, are a testament to the delicate intricacies of the female reproductive system. These benign formations, which are prevalent, are notably more common among women who are in their reproductive years and those who are nearing the menopausal phase [1]. Although their physiological impact on the body is undeniable, with symptoms ranging from pain to menstrual irregularities, their broader ramifications extend into the socioeconomic sphere. In the United States, the socioeconomic impact of UFs is stark, with an estimated financial burden escalating to $34 billion annually; the sheer scale of the problem becomes evident [2]. More worryingly, the demographic data underscore a troubling disparity. For reasons still not entirely clear, black women find themselves at the frontline of this battle, facing a disproportionately higher risk of developing UFs [3].

From a clinical perspective, the manifestations of UFs are multifaceted. Women afflicted with UFs often report a myriad of symptoms that severely impede their daily lives. Heavy menstrual bleeding (HMB) looms large and is frequently accompanied by debilitating pelvic pain, fatigue, and an unnaturally frequent urge to urinate [4]. If this was not daunting enough, UFs lay the groundwork for a plethora of complications. Anaemia, dysmenorrhea, backaches, leg pains, and even recurrent miscarriages or infertility have become real concerns for many [2].

Current medical interventions, while commendable in their intent, offer only transient respite. Surgical and radiological procedures, the predominant therapeutic strategies, are associated with their own set of complications, including the potential to undermine a woman's ability to conceive. Hormonal therapies, another popular resource, inhibit UF growth but are unsuitable for women holding desires of motherhood [5]. Consequently, there is currently a lack of long‐term noninvasive treatment options for UFs. Understanding the underlying mechanisms of tumour development is essential for developing new therapies, ideally focused on prevention rather than treatment [5, 6].

The development of UFs is intricately linked to the extracellular matrix (ECM) at both cellular and molecular levels. This complex network plays a central role in the pathogenesis of UFs. This intricate lattice, which provides structural integrity to cells, undergoes dynamic changes, which many believe hold the key to unravelling the mysteries of UFs. The interplay between cells and the ECM, particularly its aberrations, might be central to the pathogenesis of fibroids. However, the depth of this relationship remains tantalisingly out of reach, prompting our study's foray into this uncharted territory. However, the specific cellular and molecular mechanisms underlying the role of the ECM in UF development are not well defined [7]. This study aimed to investigate the role of the ECM in patient tissue‐derived myometrial stem cells (SCs) at risk (named MyoF) and on fibroid SCs (named UF), both of which were isolated from the UF‐containing uterus, and on normal myometrial stem cells (MMSCs), which were isolated from the healthy uterus (named MyoN) and cultured in a 3D organoid culture system.

Organoids are three‐dimensional structures that resemble miniaturised organs and consist of self‐organised cells derived from SCs or tissue samples [8]. Our patient tissue‐derived stem cell organoids have the potential to revolutionise biomedical research and clinical applications due to their remarkable ability to accurately replicate UF physiology and disease [9]. The utilisation of these pioneering organoids holds significant promise for recreating the intricate cellular composition and molecular characteristics of UFs [10]. Through this innovative method, researchers are afforded the opportunity to unravel the underlying role associated with UFs and the ECM [11].

The major disadvantages of 2D culture are that it does not represent cell–cell and cell–ECM interactions [12]. Additionally, UF and myometrial cells are known to quickly lose hormone responsiveness in 2D culture [13]. Thus, there is a need to find better alternative models to mimic natural cell growth and the reality of diseased tissue. Overall, the different animal models have provided relevant information regarding UF pathophysiology; however, they have faced considerable limitations. Many simple animal models do not work that well because they are too far removed from human physiology. On the other hand, experiments with more sophisticated animals such as primates are very difficult to justify ethically, in addition to high expenses [14]. Generally speaking, the 3D organoid model is superior to the 2D cell cultures by more closely mimicking in vivo tissue architecture while being more controlled, efficient, and cost‐effective than animal models.

By employing a cutting‐edge 3D organoid culture system derived from MMSCs and uterine fibroid stem cells (UFSCs), our study sought to simulate the in vivo environment while providing insight into the cellular dynamics at play. Organoids, with their ability to emulate organ structures and functions, serve as an ideal platform for this exploration. By marrying this organoid technology with RNA sequencing, we aspire to map the intricate gene expression patterns endemic to UFs, highlighting the association of the ECM and fibroid development.

## 2 Materials and Methods

### 2.1 Isolation and Purification of MyoN, MyoF and UF SCs

To accurately replicate the complexity of in vivo conditions in a 3D in vitro model, we isolated SCs from patient samples using Stro‐1/CD44 surface markers. These markers are known to specifically enrich a subpopulation of myometrial cells that exhibit the key characteristics of stem/initiating/progenitor cells. This has been previously established using fluorescence‐activated cell sorting (FACS) analysis. A detailed description of the isolation method, myometrial stem cell characteristics post‐isolation, and the assessment of stem cell marker expression was illustrated by our team's previous publication Mas, A. et al. [15]. MyoF and UF samples were obtained from women aged 18–50 years who were undergoing hysterectomy or myomectomy due to symptomatic UFs. MyoN samples were collected from women who underwent hysterectomy as a result of prolapse. Individuals with other gynaecological disorders or malignancies were excluded from the selection process. Once isolated, the SCs were cultured, maintained, and characterised in a medium comprising both Dulbecco's modified Eagle's medium DMEM and F12 supplemented with 12% foetal bovine serum (FBS) sourced from Omega Scientific (Fisher Scientific, Waltham, MA). For cultivation and expansion, these SCs were placed in flasks that were precoated with attachment factor protein from GibcoTM (Fisher Scientific).

### 2.2 Organoid 3D Culturing

Upon reaching confluence, the MyoN, MyoF, and UF SCs were dissociated using a solution of 0.25% trypsin and 0.1% EDTA in HBSS without calcium or magnesium (Fisher Scientific, Waltham, MA). The resulting cell pellet was collected through centrifugation, and the supernatant was carefully discarded. The cell pellet was subsequently resuspended in serum‐free MesenCult‐ACF Plus Medium Catalogue No# 5446 (Stemcell Technologies, Vancouver, Canada). Care was taken to disrupt any clumps through gentle pipetting, and the cell suspension was kept on ice. Cell quantification was performed using a TC20 automated cell counter (Bio‐Rad Laboratories, Hercules, CA). Approximately 10,000 cells were combined with 2 μL of Matrigel Catalogue No# 356237 (Corning, Corning, NY) yielding a final volume of 100 μL per well. The cell mixture was subsequently added to V‐bottom plates, specifically Akura 96 Spheroid Microplates Catalogue No# CS‐09‐004‐03 (InSphero), followed by centrifugation. The plates were then placed in a 37°C incubator with 5% CO_2_ for 30 min. Afterward, an additional volume of 100 μL was added to each well [13]. A 7‐day incubation period ensured the complete growth of the organoids.

### 2.3 Extended Culturing of SCs‐Derived Organoids

3D SC‐derived organoids were cultured for 21 days. The serum‐free MesenCult‐ACF Plus Medium (Stemcell Technologies, Vancouver, Canada) was weakly changed. After 21 days, the organoids were harvested, and an embedded paraffin block was generated. IHC staining of PCNA, a proliferative marker, and of Collagen type 1A (COL1A1) and Fibronectin (FN), a fibrotic markers, was compared between 3D SC‐derived organoids harvested after 7 and 21 days.

### 2.4 Organoid RNA Sequencing

First, the quality of the samples was assessed and reported by Novogene Company. Total RNA was purified to extract messenger RNA (mRNA) using poly‐T oligo‐attached magnetic beads. Subsequently, a cDNA library was constructed; the first strand of cDNA was synthesised with random hexamer primers, and then the second strand was synthesised. Library quantification was achieved using Qubit and real‐time PCR, and the size distribution was checked with a bioanalyser. The quantified libraries were pooled and sequenced on Illumina platforms based on the effective library concentration and desired data amount. The index‐coded samples were clustered according to the manufacturer's instructions, and the library preparations were sequenced on an Illumina platform, generating paired‐end reads [16].

For quality control, the raw data in fastq format underwent initial processing using fastq software. This step involved removing reads containing adapters, reads containing poly‐N sequences, and low‐quality reads from the raw data. Concurrently, the Q20, Q30, and GC contents of the clean data were calculated [17]. All subsequent analyses were performed using high‐quality clean data. The reference genome and gene model annotation files were obtained from the genome website. The HISAT2 v2.0.5 tool was used to construct an index of the reference genome, and the paired‐end clean reads were aligned to the reference genome using HISAT2 v2.0.5. Compared with nonsplice mapping tools, HISAT2 was chosen for mapping due to its ability to generate a splice junction database based on the gene model annotation file, leading to improved mapping results. The reads mapped to each gene were counted using Feature Counts v1.5.0‐p3 [18]. Subsequently, the fragments per kilobase of transcript sequence per million base pairs sequenced (FPKM) value for each gene was calculated, taking into account the gene length and the number of reads mapped to that gene. FPKM is a widely used method for estimating gene expression levels because it incorporates sequencing depth and gene length [19]. Differential expression analysis between two conditions or groups (with two biological replicates per condition) was conducted using the DESeq2 R package (1.20.0). DESeq2 utilises a model based on the negative binomial distribution to determine differential expression in digital gene expression data [20]. The resulting *p* values were adjusted using Benjamini and Hochberg's approach to control the false discovery rate. Genes with an adjusted *p* value ≤ 0.05, as determined by DESeq2, were classified as differentially expressed [21].

To analyse the functional characteristics of the DEGs, Gene Ontology (GO) enrichment analysis was performed using the clusterProfiler R package, with correction for gene length bias [22]. GO terms with a corrected *p* value less than 0.05 were considered significantly enriched for the DEGs. Additionally, the clusterProfiler R package was used to assess the statistical enrichment of differentially expressed genes in KEGG pathways, which provide insights into high‐level functions and utilities of biological systems [23]. Reactome pathways, which encompass reactions and biological pathways in human model species, were also analysed for enrichment [24].

### 2.5 Organoid Dissociation

One hundred milligrammes of papain (Cat. No. LK003176; Worthington Biochemical Corporation, USA) was reconstituted in filter‐sterilised activation solution (1.1 mM EDTA, 0.067 mM mercaptoethanol, 5.5 mM L‐cysteine HCl) to a stock concentration of 250 units/mL. The appropriate reconstitution volume was calculated by taking into account the lot‐specific % protein and activity. The solution was incubated at 37°C for 30 min to activate the enzyme. It can be stored at 2°C–8°C for up to 2 weeks. A dissociation solution was prepared (immediately before use) with 30 units/mL papain and 125 units/mL DNase I (Cat. No. EN0521; Thermo‐Scientific, Lithuania) in HBSS. The organoids were transferred to 24‐well plates, and 500 μL of dissociation solution was added. Then, the samples were incubated at 37°C for 30–60 min on an orbital shaker set to 90 rpm with intermittent trituration 5–6 times using a 1 mL pipettor at room temperature to obtain a suspension consisting primarily of single cells. The entire cell suspension was added to a 15 mL centrifuge tube containing 1 mL of 10 mg/mL ovomucoid protease inhibitor solution (Cat. No. LK003182; Worthington Biochemical Corporation, USA), and the tube was centrifuged at 300× *g* for 5 min. The cells were resuspended in the appropriate buffer (e.g., HBSS or FACS buffer), and viable cells were counted using trypan blue and a haemocytometer. The cell suspension was passed through a 37 μm strainer, and the flow‐through was retained to remove the remaining cell aggregates. The resulting single‐cell suspension was used for antibody labelling for flow cytometry [25, 26, 27].

### 2.6 Organoid Real‐Time PCR

Real‐time PCR, also known as quantitative polymerase chain reaction (qPCR), is a robust molecular technique utilised to quantitatively measure and analyse gene expression levels. For RNA extraction, the miRNeasy Tissue/Cells Advanced Micro Kit Cat. No. 217684 (Qiagen, Valencia, CA) was used according to the manufacturer's instructions. Reverse transcription was conducted using the RNA to cDNA EcoDry Premix (Double Primed) Kit, *Cat. No. 639549* (Takara Bio, USA). RT–PCR was performed on a CFX Connect RT–PCR Detection System (Bio‐Rad Laboratories, Hercules, CA) using Advanced Universal SYBR Green qPCR Master mix (Takara, Tokyo, Japan).

The proliferation markers, including Cyclin D, as well as the expression levels of TGF‐β3, COL1A1, COL3A1, and FN, key components of the ECM, were analysed in the organoids.

The PCR primers used in the experiment can be found in the provided Table S1, and the expression levels were normalised to those of GAPDH in each patient. The PCR amplification procedure included an initial predenaturation step at 95°C for 30 s, followed by 40 cycles of denaturation at 95°C for 15 s, annealing at 60°C for 30 s, and extension at 65°C for 31 s. The relative expression of the target gene was calculated using the 2^(−ΔΔCT)^ method [28].

### 2.7 Organoid Paraffin Block Formation and Immunohistochemical (IHC) Staining

MyoN, MyoF, and UF organoids, along with myometrial/UF tissue samples from corresponding patients, were submitted to the Organoid and Primary Culture Research Core at the University of Chicago for processing. Paraffin‐embedded organoid blocks were created within the organoid core using established procedures. Briefly, SCs‐derived organoids were collected in a microcentrifuge tube and fixed in 10% paraformaldehyde for 60 min. Then, 1% agarose solution (Agarose I, VWR Corporation, USA) was added, mixed gently with the organoids, and allowed to solidify for approximately 1 h at room temperature. The agarose gels of the organoids were sliced longitudinally and processed on a tissue processor (Leica ASP6025, Germany) before embedding in paraffin. Sections were cut at 5 μm for haematoxylin and eosin (H&E) and Masson's trichrome staining. Masson's trichrome was used to stain the collagen blue, and smooth muscle (fibroid cells) was stained red. The tissue structure and (ECM) were visualised. Immunohistochemical (IHC) staining was performed at the University of Chicago Pathology Core Facility. Proliferation markers, including PCNA and Cyclin D, were investigated to assess cellular proliferation within the organoids [29]. Antibody information is summarised in the provided Data S1. The expression levels of β‐catenin, periostin, COL1A1, COL3A1, and FN were also analysed to assess the composition of the ECM [30, 31]. The slides were scanned and analysed using the Aperio ImageScope colocalization algorithm—Pathology Slide Viewing Software. These investigations yielded valuable insights into the cellular and molecular characteristics of the organoids, shedding light on aspects related to tissue structure, cell proliferation, and the ECM.

### 2.8 Frozen Sectioning and Immunofluorescence Staining

For cryosectioning, the organoids were first transferred to 1.5 mL tubes and centrifuged, after which the pellets were washed with PBS. The organoids were subsequently fixed with 4% paraformaldehyde for 60 min at 4°C and washed again with PBS. The organoids were then resuspended in 2% methylene blue for staining for 20 min, washed with PBS, and cryoprotected overnight by suspension in 30% sucrose. The next day, the sucrose was removed by centrifugation, and the organoids were embedded in an OCT compound (Fisher Scientific, Waltham, MA) and flash‐frozen at −80°C. The organoids embedded in OCT were sectioned using a cryostat (Thermo Scientific Microm HM 550, Fisher Scientific, Waltham, MA). The sections were preserved at 4°C for staining. The staining was performed according to the following method.

The slides were washed 3 times with PBS. After washing, 400 μL of 0.1% Triton in PBS was added for 15 min at room temperature. The organoids were blocked with 5% BSA in PBS for 60 min and incubated overnight with primary antibodies Table S2. The organoids were washed in PBS three times and then incubated for 1 h in the dark with Alexa Fluor‐labelled fluorescent dye secondary antibodies (1:1000; Life Technologies, CA, USA). After washing with PBS twice, the organoids were mounted in VECTASHIELD Antifade Mounting Medium supplemented with DAPI (1 ng/mL, Vector Laboratories, CA, USA). Images were acquired using an Olympus BX41 microscope (Olympus America, Center Valley, PA) at the University of Chicago Integrated Light Microscopy Core. The antibodies used in this study are listed in Table S2. The images were analysed by QuPath software for bioimaging analysis.

### 2.9 Immunofluorescence Staining of Whole‐Mount Organoids Using Antibodies

The organoids were washed twice with PBS (1X) (pH 7.4; Cat. No. 70011; Gibco, USA). The organoids were fixed with 4% PFA (Cat. No. FB002; Invitrogen, USA) for 60 min at room temperature. Then, the organoids were washed with PBS three times. The fixed organoids were permeabilised with 0.5 mL of blocking buffer (5% horse serum + 0.5% Triton X‐100 in 1X PBS) overnight at 4°C. The blocking buffer was removed, primary antibodies were added (300–500 μL), and the samples were incubated at 4°C overnight. Antibody information is summarised in Table S2. The organoids were washed three times with PBS, and the membranes were incubated with secondary antibodies overnight at 4°C. For nuclear staining, organoids were incubated with 5 μg/mL DAPI (1 mg/mL, Cat. No. 62248; Thermo Scientific, Germany) in 1× PBS (300–500 μL per sample) at room temperature for 20 min. Then, the organoids were washed three times with PBS and imaged on a confocal microscope (2‐photon Leica Microscope) at the University of Chicago Integrated Light Microscopy Core. The images were analysed by QuPath software for bioimaging analysis.

### 2.10 Flow Cytometry of the Dissociated Organoid to Confirm Its Cellular Components

The cells were first blocked with sterile 5% BSA in PBS and incubated for 30 min on ice. After blocking, the cells were washed with 500 μL of sterile PBS and then centrifuged at 1500 RPM for 5 min at 25°C. The supernatant was aspirated, and either 500 μL of sterile 3% BSA in PBS (as a control) or a specified volume of the primary antibody dissolved in 3% BSA in PBS was added. Information regarding the antibodies used is detailed in Table S2. The tubes were incubated on ice for 1 h in the dark. Following this incubation, the tubes were aspirated, and the secondary antibody was added to 3% BSA in PBS. Approximately 10–15 min before the endpoint, 5 μL of DAPI was added to the tubes (to sort only viable cells), and the plates were incubated at room temperature in the dark. After this, the tubes were washed with sterile PBS and centrifuged at 1500 RPM for 5 min at 25°C. Then, 500 μL of 3% BSA in PBS was added to each tibia, the mixture was mixed well, and the contents were transferred to labelled FACS tubes (Falcon #352058) for analysis via Fortessa 4–15 HTS.

### 2.11 Mechanical Characterisation of SCs‐Derived Organoids

Organoids were tested with a single indentation protocol using a Piuma nanoindenter (Optics11, Amsterdam, Netherlands). Subsequent nanoindenter testing was performed on Myo N, Myo F, and UF SC‐derived organoids from the uterine tissue of White and Black patients. The Hertz method was used for analysis because the use of the loading portion of the load–displacement curve is far more suitable for soft biological samples [32]. The Hertz model assumes a linear elastic material response. The elastic Young's modulus was calculated according to Rachel et al. 2021 [32].

### 2.12 Measurement of DNMT Activity

The organoids were subjected to nuclear extraction using an EpiQuik Nuclear Extraction Kit (Cat. No. #OP‐0002; Epigentek, NY, USA) according to the manufacturer's instructions. The protein concentration in the nuclear extract was measured using a protein assay dye reagent concentrate kit (Bio‐Rad Laboratories, USA) (Cat. No. #5000006). DNMT activity was measured according to the *manufacturer's* instruction manual of the Colorimetric ELISA Easy Kit, Cat. No. #P‐3139‐96 (Epigentek, NY, USA).

### 2.13 Assessment of Viability and Apoptosis

Viability assessment was conducted using the CellTiter‐Glo 3D Cell Viability Assay (Promega, Germany), which is specifically designed for determining cell viability in 3D organoids. This assay utilises ATP as an indicator of viability and generates a highly sensitive luminescent readout, surpassing the capabilities of colorimetric or fluorescence‐based methods in culture media. To initiate the assay, an equivalent volume of CellTiter‐Glo 3D Reagent to the volume of the cell culture medium present in each well was added. The contents were vigorously mixed for 5 min to induce cell lysis. Subsequently, the plate was incubated at room temperature for an additional 25 min. This stabilised luminescent signal was then recorded as a measure of viability.

Similarly, RealTime‐Glo Annexin V Apoptosis and Necrosis Assay (Promega, Germany) was performed. This assay is designed to provide real‐time and kinetic measurements of apoptosis and necrosis in live cells without cell lysis. It specifically detects the exposure of phosphatidylserine (PS) on the outer leaflet of the cell membrane, which is a well‐established and validated indicator of apoptosis. The assay utilises annexin V binding to detect PS exposure, and this binding event is detected using a luminescent signal.

### 2.14 Using Organoids as a Tool for Testing the Effect of Different Hormonal Exposures

Organoids were cultured from SCs derived from tissue at risk (MyoF) and uterine fibroid tissue (UF) from subjects with UFs. After 7 days of culture, the organoids were washed twice with Dulbecco's phosphate‐buffered saline (DPBS). Subsequently, the cells were exposed to four different media: DMEM/F12 phenol red‐free media supplemented with insulin‐transferrin‐selenium (ITS) from Gibco and human recombinant EGF (epidermal growth factor) from Millipore, which served as the untreated control; estradiol (E2) Cat. No. 50‐28‐2 (Millipore Sigma, USA), which was dissolved in treated media at a concentration of 10 ng/mL; progesterone (P4) Cat. No. S1705 (Selleckchem, TX, USA), which was dissolved in treated media at a concentration of 10 ng/mL; and a combination of oestrogen and progesterone at a concentration of 10 ng/mL each. Then, the cells were incubated in a 37°C incubator with 5% CO_2_ for 48 h. After 48 h, the organoids were collected, washed, and sent for paraffin‐embedded organoid block formation, and then subjected to further IHC staining for oestrogen receptor, PCNA, and BCL‐2 in the control and treated organoids.

### 2.15 Using Organoids as a Tool to Study Uterine Fibroid Health Disparity

Organoids were cultured from SCs derived from various sources: a black normal subject (MyoN B), a white normal subject (MyoN W), MMSCs derived from tissue at risk in black subjects with UFs (MyoF B), and tissue at risk in white subjects with UFs (MyoF W). The culturing was carried out according to a previously described method.

Further investigations were conducted specifically on MyoF from both White and Black subjects. These included IHC staining for Periostin and COL1A1, trichrome staining, and mechanical characterisation assessments for all groups.

### 2.16 Using Organoids as a Tool for Drug Screening

Organoids were cultured from SCs derived from tissue at risk (MyoF) and uterine fibroid tissue (UF) from subjects with UFs. After 7 days of culture, the organoids were washed twice with DPBS. Subsequently, UF organoids were exposed to 10or 100 nM vitamin D3 and 10 or 100 nM Doxercalciferol for up to 15 days.

Further investigations were conducted, including assessment of viability and apoptosis; IHC staining for BCL‐2, IRF‐3, NF‐κβ and CXCR4; and trichrome staining for all groups.

On the other hand, MyoF organoids were exposed to 100 nM vitamin D3, green tea extract Epigallocatechin Gallate (EGCG) or their combination for 48 h. Further investigations, including mechanical characterisation assessments and DNMT activity measurements, were conducted for all groups.

### 2.17 Using Organoids as a Tool to Study the Pro‐Fibroid Effects of Environmental Phthalates

Organoids were cultured from SCs derived from normal myometrium (MyoN), and culturing was carried out according to a previously described method. After 7 days of culture, the organoids were washed twice with DPBS. Subsequently, the plants were subjected to two different treatments: Mono‐butyl phthalate, Cat. No. 75958 (Sigma–Aldrich, Germany) (MBP), and di‐butyl phthalate, Cat. No. 84742 (Sigma–Aldrich, Germany) (DBP), at concentrations of 0.16 and 1.6 μM, respectively. In addition, dimethyl sulfoxide (DMSO), sterile filtered Cat. No. 3176 (Tocris Bioscience, Canada), which was used as a vehicle to dissolve MBP and DBP and served as the untreated control, was added to DMEM/F12 phenol red‐free media supplemented with ITS from Gibco and human recombinant EGF (epidermal growth factor) from Millipore. Further investigations, including assessments of viability and apoptosis, were conducted.

### 2.18 Statistical Analysis

All the raw data were collated in a Microsoft Excel database, and Prism 9 was used for the statistical analysis. The data are presented as the mean ± SD or mean ± SEM (standard error of the mean). The statistical significance of the differences was determined by *t*‐tests, one‐way or two‐way analysis of variance (ANOVA), followed by Tukey's test. A *p* value less than 0.05 in a two‐tailed analysis was considered to indicate statistical significance.

## 3 Results

### 3.1 Development of a 3D Stem Cell (SCs)‐Derived Organoid Culture Model

**FIGURE 1:**
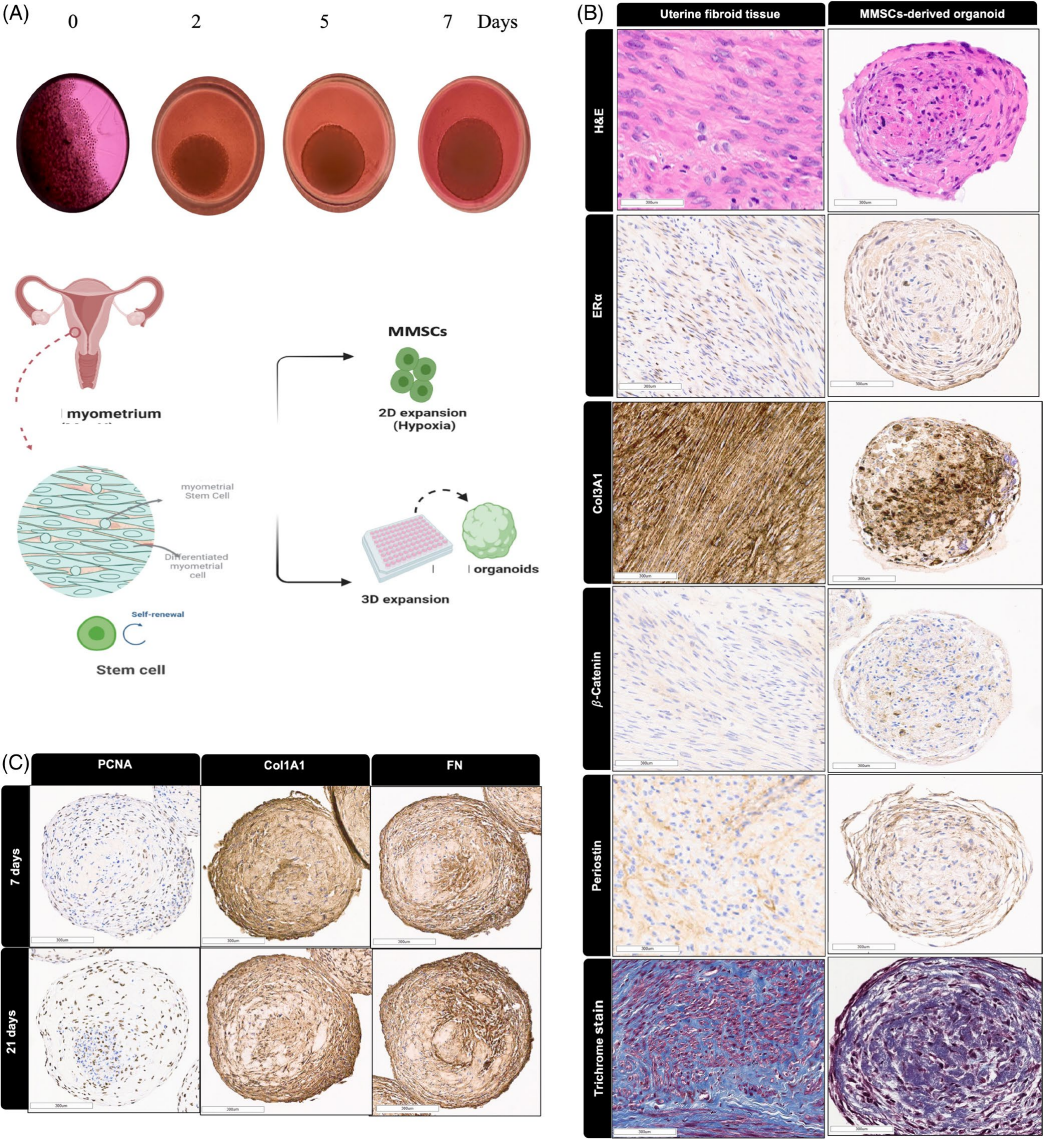
(A) Representative phase images demonstrating the development of SCs‐derived organoids in vitro. (B) Morphology and IHC staining of 3D SCs‐derived organoids and their original primary human tissues. Haematoxylin and eosin (H&E) staining (top) and staining for the oestrogen receptor (ERα), Col3A1, β‐Catenin, periostin, trichrome stain (bottom) are shown. (C) Morphology and IHC staining of 3D SCs‐derived organoids cultured for 7 or 21 days. PCNA (left), COL1A1 and fibronectin (right). Scale bar = 300 μm. All the data are from five independent (*n* = 5) experiments performed for each individual group. The slides were scanned and analysed using the Aperio ImageScope colocalization algorithm—Pathology Slide Viewing Software.

One of the primary advantages of 3D culture systems over traditional 2D systems is their enhanced ability to mimic in vivo tumour environments. As early as the first day post‐seeding, SCs began to self‐aggregate, forming organoids, as depicted in (Figure 1A). We determined the optimal seeding density to be 10,000 SCs, which consistently formed organoids when cultivated in a defined culture system. This system employed Matrigel as an ECM scaffold for the embedded cells and MesenCultTM‐ACF Plus Medium for 7 days in an ultralow attachment 96‐well plate (Figure 1A). In this setting, cells self‐organised by the first day, evolving into organoid structures. Over the course of 7 days, these structures exhibited exponential growth in both size and cell count (Figure 1A).

Compared to UF tissue, 3D organoids closely mirrored the morphology observed in vivo in fibroids and were primarily composed of characteristic smooth muscle cells and ECM (Figure 1B). In the 3D organoids, there were noticeable deposits of trichrome stain, Collagen 3A1 (COL3A1), periostin, as they are ECM markers that have been explored in UF, ‐catenin, since it is involved in UF pathogenesis and Oestrogen Receptors (ERα) as this isa hormonal dependent tumour. This deposition resembled the prominent collagen accumulation characteristically observed in human UF tissues. Collectively, these observations suggest that arranging cells in a 3D matrix fosters a microenvironment conducive to maintaining cell vitality as ERα responds to hormones as well as‐catenin signalling that enhances UF proliferation [33]. This, in turn, prompts both cell growth and ECM production, features routinely observed in vivo UFs.

### 3.2 Effect of Extended Culturing of 3D Stem Cell (SCs)‐Derived Organoids

3D SCs‐derived organoids were sustainably maintained in culture for up to 3 weeks. When comparing the IHC results between organoids cultured for 7 days and those cultured for 21 days, we noticed that organoids still express essential markers characteristic of UF, such as PCNA as a proliferation marker and ECM markers such as COL1A1 and FN, which remained consistent across both time points (Figure 1C).

### 3.3 Derivation of Myometrial Organoids From CD44/Stro1 SCs and Their Differentiation Under Normoxic Conditions

**FIGURE 2:**
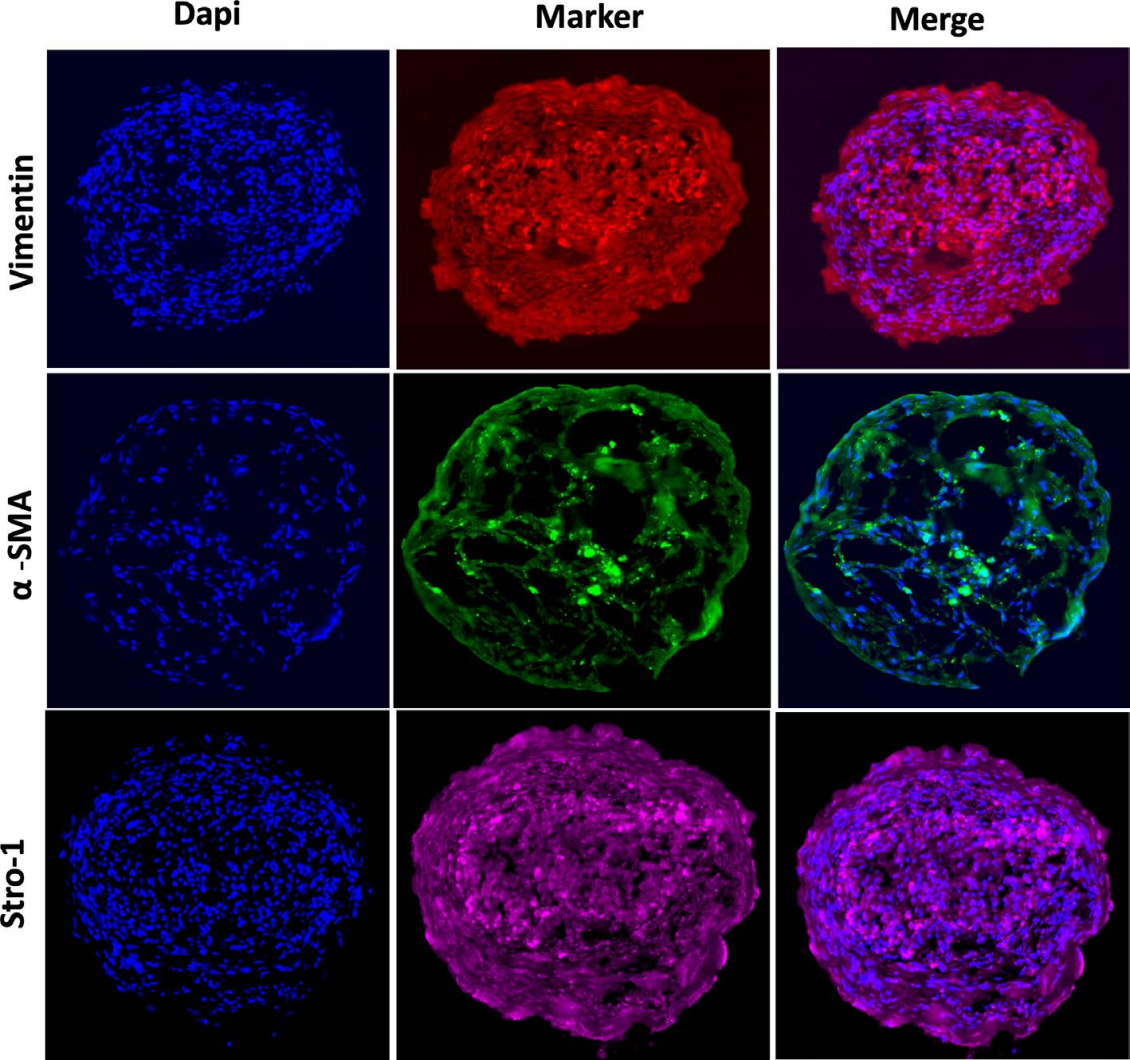
Identification of 3D SCs‐derived organoid cellular content. On day 7, the organoids were cryosectioned, and immunostaining was performed for vimentin (red) (top), alpha smooth muscle Actin α‐SMA (green) and Stro‐1 (magenta). Representative confocal images demonstrating the differentiation of SCs into different cell types in the organoids. The nuclei were counterstained with 4′,6‐diamidino‐2‐phenylindole (DAPI, blue).

As mentioned in the Materials and Methods section, we used a subpopulation of myometrial SCs with Stro1/CD44 surface markers enriched in SCs. Stro‐1/CD44 SCs were isolated from human myometrial and uterine fibroid biopsies using enzymatic digestion and flow cytometry. We analysed SCs‐derived 3D organoids under normoxic conditions in MesenCultTM‐ACF Plus Medium by immunofluorescence and flow cytometry. By immunofluorescence, our organoids expressed stromal (mesenchymal) cells marker (Vimentin), smooth muscle cells marker α smooth muscle actin (α SMA) and SCs marker as Stro‐1 (Figure 2). After the dissociation of the organoids, the relative percentage of live cells was 95.8%, as determined by DAPI staining (Figure S1). By flow cytometry, the dissociated organoids express 37.9% of the cells expressed dual surface markers (CD44/Stro1), 7.7% express stromal (mesenchymal) cells markers (Vimentin) and 50.7% express smooth muscle cells marker (α SMA) (Figure S1).

**FIGURE 3:**
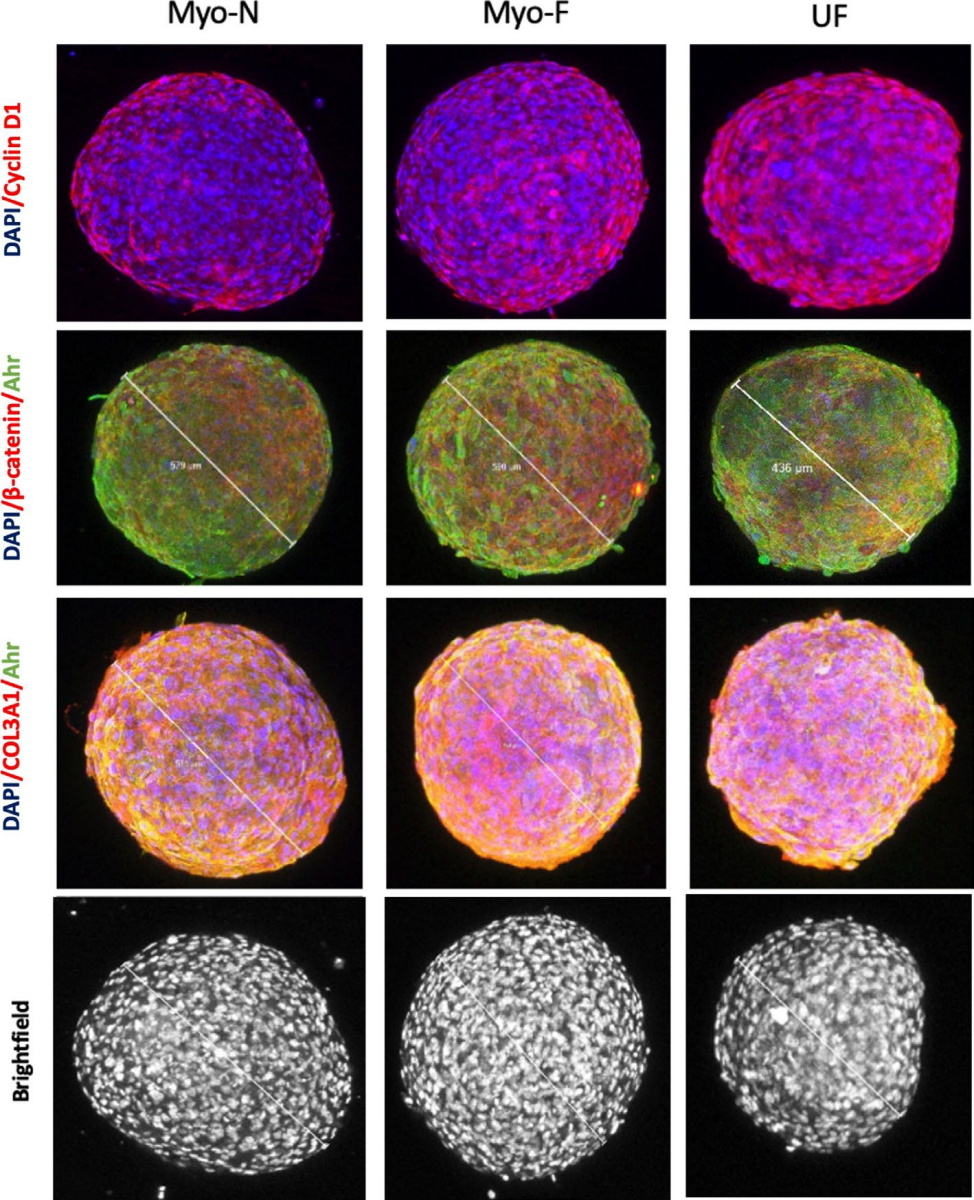
On day 7, representative confocal images of whole intact MyoN, MyoF and UF organoids were obtained, and immunofluorescence staining was performed for CyclinD1 (top), β‐catenin, Col3A1, Ahr (green) and the bright field (bottom). The nuclei were counterstained with 4′,6‐diamidino‐2‐phenylindole (DAPI, blue). Organoids were imaged on a 2‐photon Leica Microscope. All the data are from three independent (*n* = 3) experiments performed for each individual group. The images were analysed by QuPath software for bioimaging analysis.

Additionally, the organoids expressed an ECM marker (COL 3A1), a proliferative marker (cyclin D1), the total epithelial‐mesenchymal transition marker EMT (β‐catenin) and aryl hydrocarbon receptor (AHR) (Figure 3) as AHR pathway activation has a role in UF survival [34].

### 3.4 RNA‐Seq Analysis Revealed a Disrupted ECM Accumulation

RNA‐Seq analysis was performed on 3 comparisons, namely, MyoF –MyoN, UF–MyoF, and UF–MyoN. Biological replicates (*n* = 3) were used to ensure experimental validity. Evaluating gene expression correlations between samples helped verify the reliability and proper sample selection, aiding in differential gene expression analysis. The correlation coefficient, a measure of sample similarity, was critical for assessing data quality, and an ideal experimental condition required a coefficient > 0.92 and R2 > 0.8. Differentially expressed genes from the comparison groups were combined to create the differential gene set.

**FIGURE 4:**
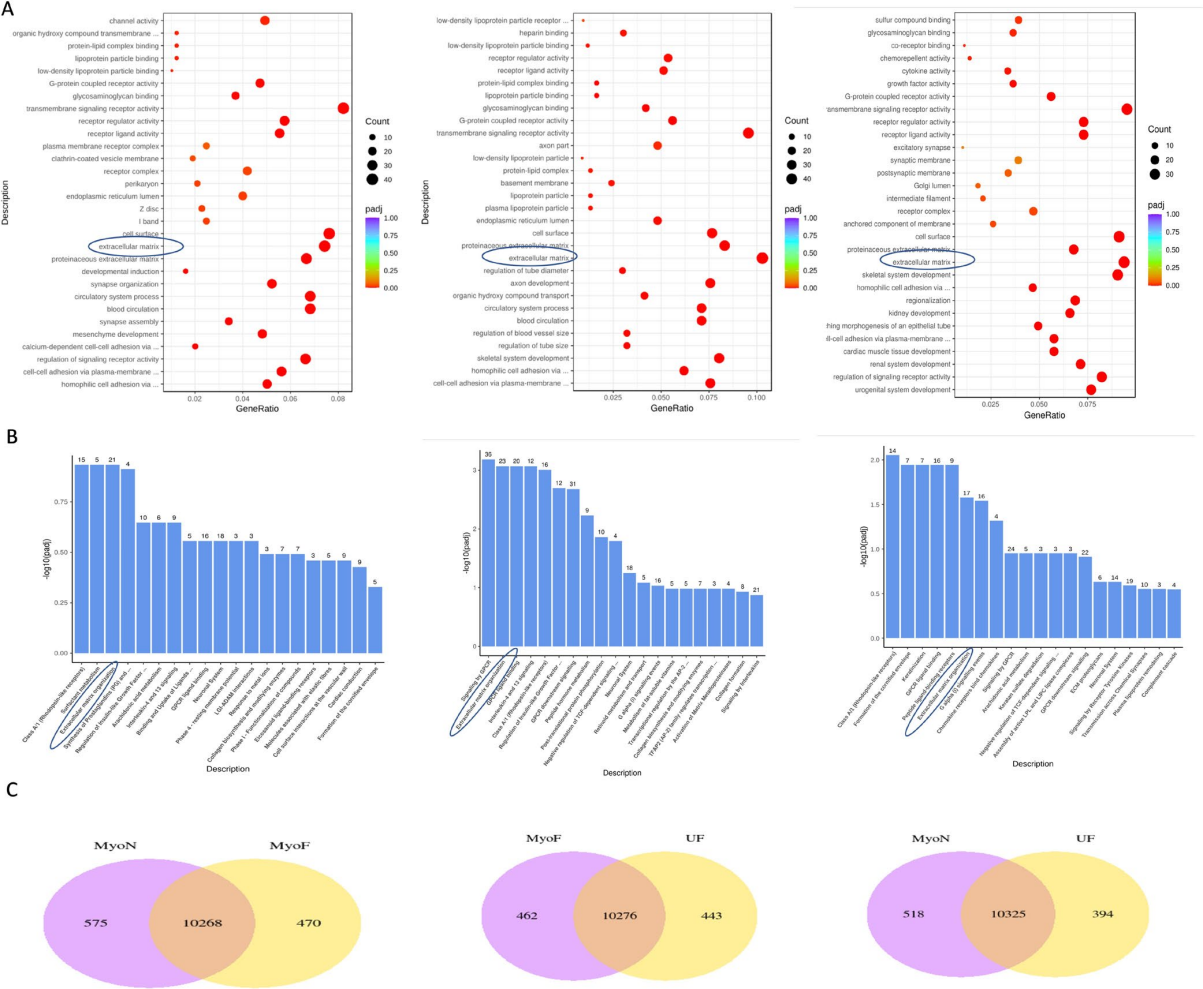
RNA sequencing revealed the role of ECM‐related pathways in the transition from normal to at risk myometrium to UFs. (A) Bubble chart of GO enrichment analysis for overrepresented analysis of MyoF over MyoN, UF over MyoF and UF over MyoN (*n* = 3). (B) Reactome pathway analysis of MyoF over MyoN, UF over MyoF and UF over MyoN (*n* = 3). (C) Venn diagrams illustrating the overlap of differentially expressed genes in MyoF over MyoN, UF over MyoF and UF over MyoN (*n* = 3).

In this study, based on the results, we conducted a comprehensive analysis by focusing on three experimental groups, each representing a specific comparison. These groups were named MyoF‐MyoN, UF‐MyoF, and UF‐MyoN. In each comparison, we investigated the gene expression patterns to gain insights into the underlying biological processes (BP) and pathways. In the first comparison, we looked at the MyoF group versus the MyoN group, UF versus MyoF, and UF versus MyoN. Up to 30 ECM genes and proteinaceous ECM genes were found to be significantly different between the MyoF group versus the MyoN group, UF versus MyoF, and UF versus MyoN. There were other genes differentially expressed between groups, but in this manuscript, we focused on ECM. The co‐expression Venn diagram visually depicted the genes that were exclusively expressed within each group and those that were co‐expressed in multiple groups. According to the GO enrichment dot analysis for BP, the ECM exhibited statistical significance (Figure 4A). Reactome pathway analysis also highlighted the significance of the ECM as well (Figure 4B). Notably, the groups shared 10,268 genes, 10,276 genes, and 10,325 genes, respectively (Figure 4C).

### 3.5 Quantitative PCR Showing Increased Expression of ECM Related Markers in UF Organoids

**FIGURE 5:**
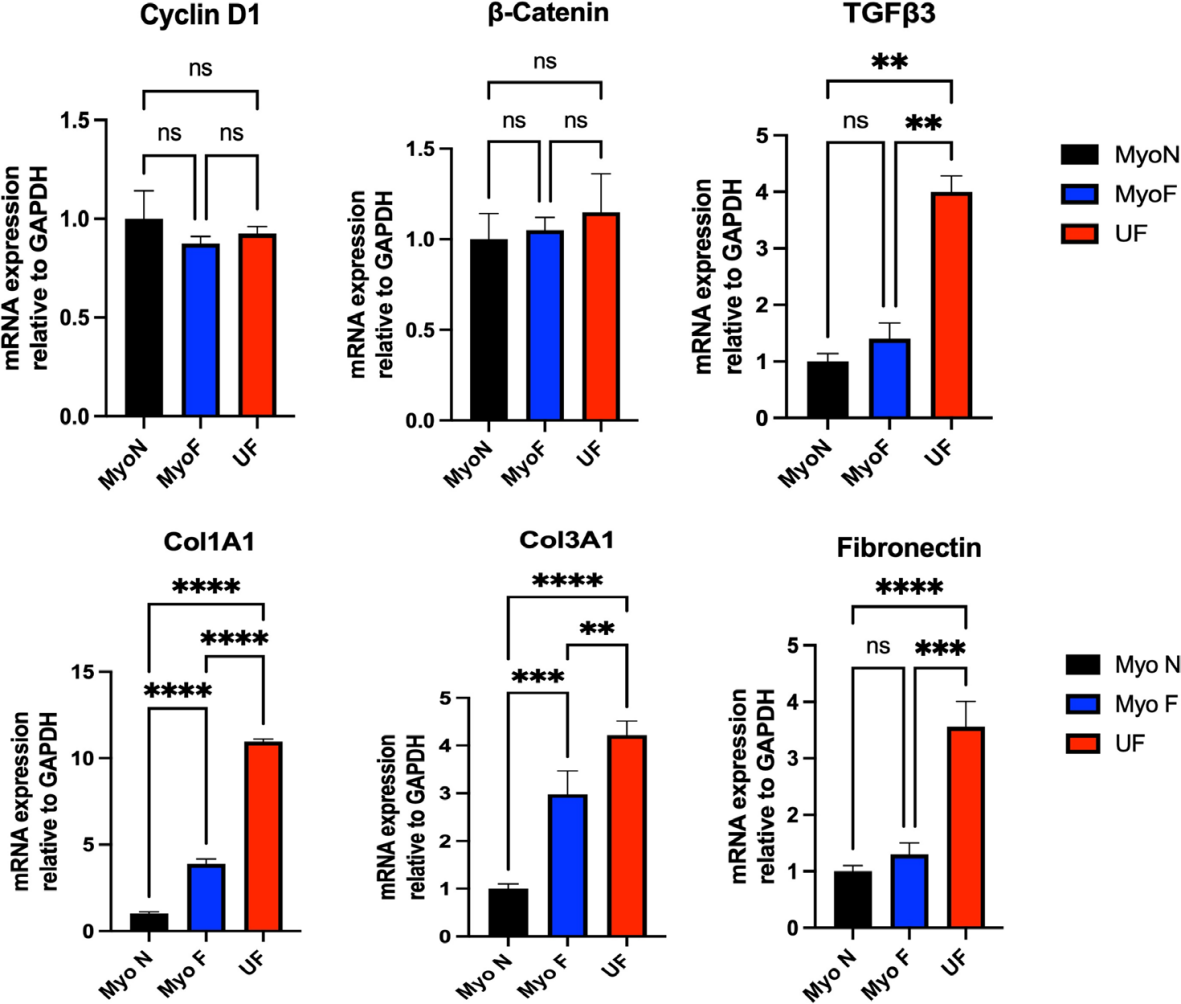
The relative changes in the gene expression of *Cyclin D1* (top, left), *β‐catenin*, *TGFβ‐3* (top, right), *COL1A1* (bottom, left), *COL3A1*, and Fibronectin (bottom, right) in MyoN, MyoF, and UF organoids were quantified by the 2 −ΔΔCT method. *, **, and *** indicate statistical significance according to two‐way ANOVA with Tukey's post hoc test (*p* < 0.05, *p* < 0.01, and *p* < 0.001); ns: Nonsignificant. The bar graphs represent the mean ± SEM of three technical replicate measurements of three independent biological replicates from three different patients.

To validate the RNA seq findings regarding ECM disruption, RNA was isolated from 7‐day cultured normal myometrial, myometrium at risk, and fibroid organoids, and qRT–PCR analysis revealed that there was a significant increase in the expression of several ECM markers, including *COL1A1*, *COL3A1*, and *FN1*, derived from UF, with 10‐fold, 3‐fold, and 2‐fold increases, respectively, compared to SCs derived from the uterus without UFs (MyoN). However, the levels of *COL1A1* and *COL3A1* significantly increased 3‐fold and 2‐fold, respectively, in the organoids derived from MyoF SCs (*p* < 0.001) (Figure 5). Additionally, UF organoids showed a significant 3‐fold increase in *TGFβ‐3* relative to MyoN (*p* < 0.01). On the other hand, no significant difference in *Cyclin D1* or *β‐Catenin* expression was detected between the MyoN‐, MyoF‐, or UF‐derived organoids (Figure 5). As a conclusion of this experiment, there is a significant increase in ECM‐related markers and *TGFβ‐3* in MyoF and UF organoids relative to normal myometrium organoids.

### 3.6 Measurement of DNMT Activity

There is some new area of treatments under investigation targeting DNMT activity, so our measurement of DNMT activity opens a new area of research to discover some suitable treatments in the future. By measuring the total DNMT activity of the three organoids, we detected a significant increase in the DNMT activity of MyoF relative to that of MyoN and UF (*p* < 0.05) (Figure S2).

### 3.7 Immunohistochemistry Shows Increased Protein Expression of ECM Related Markers in UF Organoids

**FIGURE 6:**
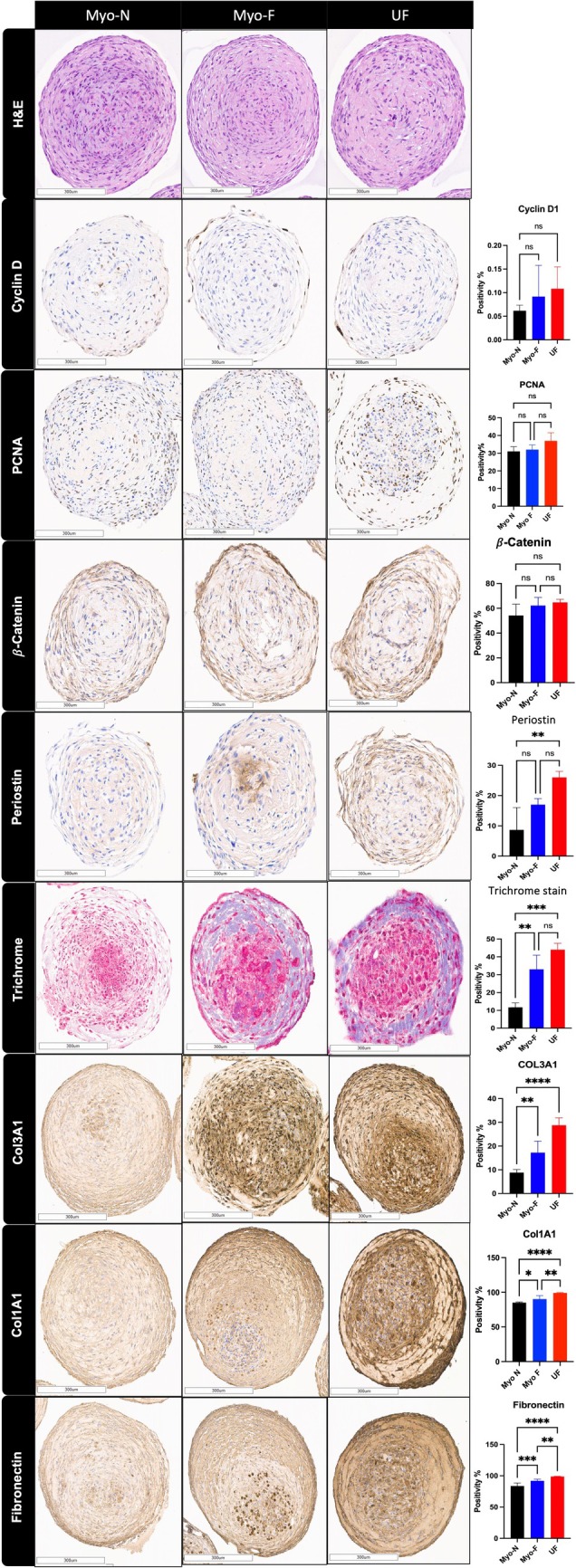
Histology and immunohistochemical staining of MyoN, MyoF, and UF organoids; haematoxylin and eosin (H&E) staining (top); Cyclin D1, β‐Catenin, Periostin, Masson's Trichrome staining; and COL1A1, COL3A1, and Fibronectin (bottom). Scale bar = 300 μm. The slides were scanned and analysed using the Aperio ImageScope colocalization algorithm—Pathology Slide Viewing Software *, **, and *** indicate statistical significance according to two‐way ANOVA with Tukey's post hoc test (*p* < 0.05, *p* < 0.01, and *p* < 0.001); ns: nonsignificant. The bar graphs represent the mean ± SEM of three technical replicate measurements of three independent biological replicates from three different patients.

For validation of RNA seq findings of ECM disruption on protein level, we assessed the expression of ECM‐related proteins, including periostin, COL1A1, COL3A1, and FN, by immunohistochemistry of paraffin‐embedded organoids as well as Masson's trichrome stain. ECM proteins were upregulated in UF organoids compared to MyoN organoids. The periostin level was significantly greater by 18% in UF organoids than in MyoN organoids (*p* < 0.01). The percentage of trichomes stained with both MyoF and UF was 21% and 33%, respectively, greater than that stained with MyoN (*p* < 0.01, *p* < 0.001). Similarly, compared with MyoN, MyoF exhibited high levels of COL1A1, COL3A1, and FN (6%, 9%, and 10%, respectively) (*p* < 0.05, *p* < 0.01, *p* < 0.001). Additionally, compared with MyoN, UF significantly increased the expression of COL1A1, COL3A1, and fibronectin by 11%, 21%, and 15%, respectively (*p* < 0.0001) (Figure 6). For the proliferative markers, no significant difference was detected in Cyclin D1, PCNA, and β‐catenin between MyoN, MyoF, and UF 3D SCs‐derived organoids (Figure 6). As a conclusion of this experiment, there is a significant increase in ECM‐related markers in UF organoids relative to normal myometrium organoids.

### 3.8 Mechanical Properties of SCs‐Derived Organoids

**FIGURE 7:**
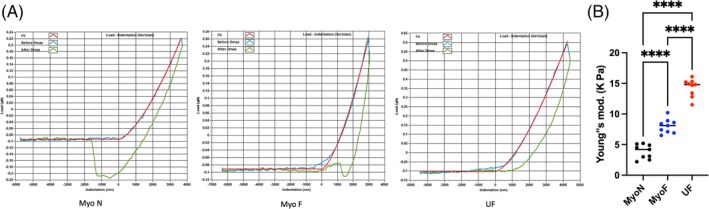
The mechanical properties of SCs‐derived organoids were tested with a single indentation protocol using a Piuma nanoindenter (Optics11, Amsterdam, Netherlands). (A) An exemplary force curve obtained from nanoindentation testing of SCs‐derived organoids. Magnification of the force curve demonstrates the smoothing effect of the Hertzian curve fit to the raw data. (B) Young's modulus of Myo‐N‐, Myo‐F‐, and UF SCs‐derived organoids. *, **, and *** indicate statistical significance according to two‐way ANOVA with Tukey's post hoc test (*p* < 0.05, *p* < 0.01, and *p* < 0.001); ns: Nonsignificant. Each biological replicate is shown as a *circle* and represents the average of five technical replicate measurements.

Figure 7 displays a schematic representation of the force curves obtained from stiffness measurement of SCs‐derived organoids using the Piuma nanoindenter. The smoothing effect of the Hertzian curve fit to the raw data is evident. As shown in Figure 7, the elastic Young's modulus increased over time. However, the rate of this increase varied across the three organoid types. Specifically, the Young's modulus of UF organoids was significantly different from that of MyoF and MyoN, indicating that UFs were stiffer, with a twofold increase relative to that of the normal myometrium. In comparison, the elastic Young's modulus of MyoF organoids (those considered at risk) was notably greater (by a factor of one‐fold) than that of MyoN organoids but was significantly lower than that of UFs. These findings highlight the potential role of mechanotransduction as a contributing factor to the uterine fibroid formation process.

### 3.9 Organoids as a Tool for Testing the Effect of Different Hormonal Exposures

**FIGURE 8:**
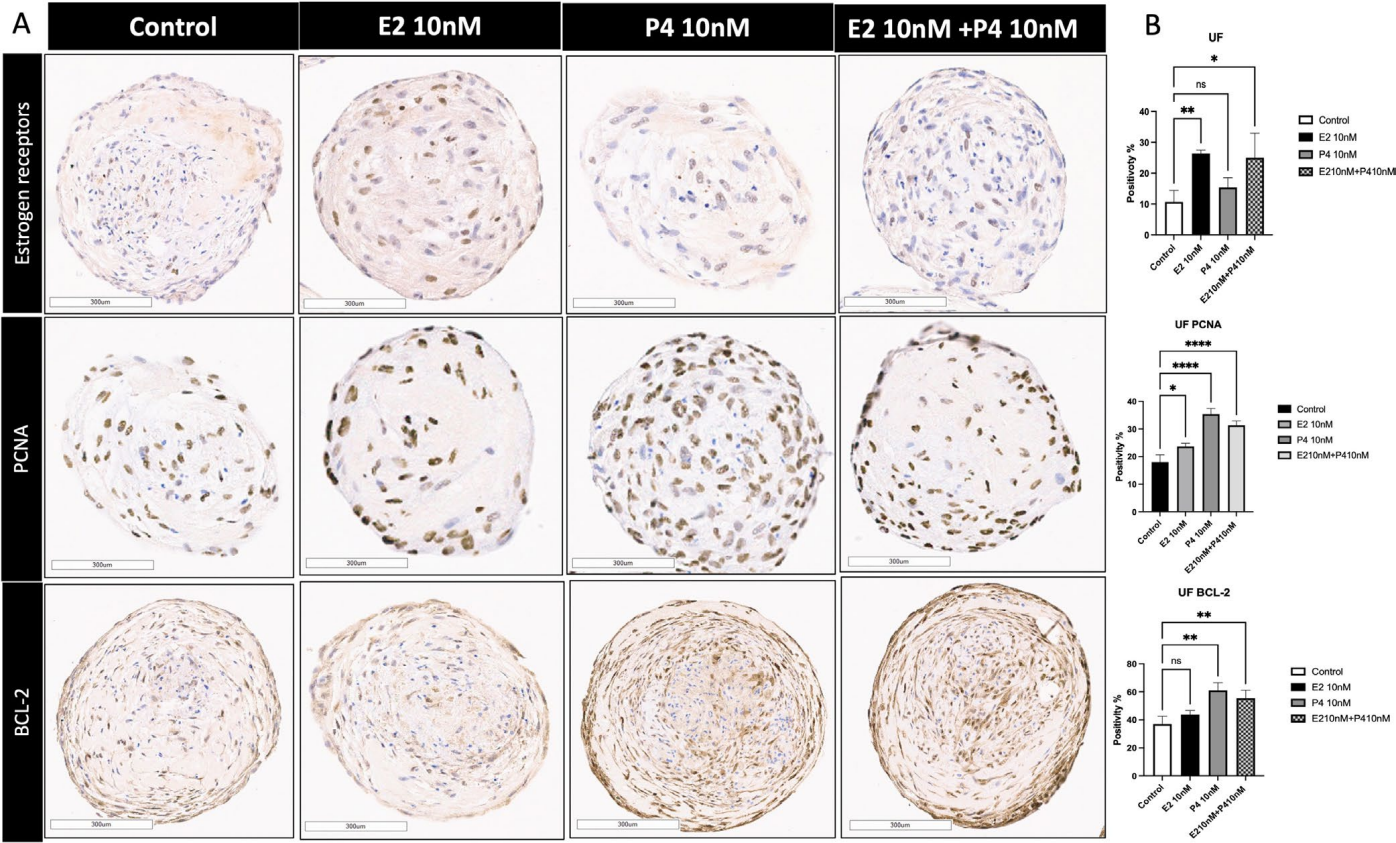
Response of SCs‐derived organoids treated with estradiol (E2, 10 ng/mL), progesterone (P4, 10 ng/mL) or their combinations to ovarian steroid hormones for 48 h. (A) Immunohistochemical staining for the oestrogen receptor, PCNA and BCL‐2 in UF SCs‐derived organoids. Scale bar = 300 μm. (B) The bar graph is a quantitative representation of the IHC staining of the ER antigen, PCNA and BCL‐2 on the SCs‐derived UF organoids in response to E2 and P4. The slides were scanned and analysed using the Aperio ImageScope colocalization algorithm Pathology Slide Viewing Software. *, **, and *** indicate statistical significance according to two‐way ANOVA with Tukey's post hoc test (*p* < 0.05, *p* < 0.01, and *p* < 0.001); ns: Nonsignificant. The bar graphs represent the mean ± SEM of five technical replicate measurements of three independent biological replicates from three different patients.

The human uterine myometrium is influenced by ovarian steroid hormones. To investigate the hormone responsiveness of the organoids in vitro, the myometrial organoids were treated on day 7th with 10 ng/mL oestrogen (E2), progesterone (P4) or their combination for 48 h. After the treatment period, the organoids were harvested and subjected to IHC staining for oestrogen receptor (ERα), PCNA, and BCL‐2. Our study sought to elucidate the response of MyoF‐ and UF SCs‐derived organoids to the ovarian steroid hormones E2 and P4. The stained slides were scanned and analysed using the Aperio ImageScope colocalization algorithm Pathology Slide Viewing Software. Observations of UF‐derived organoids revealed a marked and significant increase in ER antigen expression in both the estradiol‐ and combination‐treated groups compared to that in the control group, with increases of 15.6% and 14.3%, respectively (*p* < 0.01, 0.05). In contrast, the MyoF‐derived organoids demonstrated significant increases in ER antigen expression in both the estradiol‐treated group and the combination‐treated group relative to the control group, with increases of 14.34% and 9.64%, respectively (*p* = 0.001, 0.01) (Figures 8 and S3). The level of PCNA, a proliferative marker, increased significantly in both the progesterone‐ and combination‐treated groups in both the MyoF (6.7%, 8.3%) and UF (17.3%, 13.3%) 3D organoid (*p* < 0.05, 0.01, 0.0001, 0.0001) groups. BCL‐2, an antiapoptotic marker [35], was significantly increased in both the progesterone‐ and combination‐treated groups in both the MyoF (8.3%, 7%) and UF (24%, 18.3%) 3D organoid groups (*p* < 0.05, 0.01, 0.0001, 0.0001).

### 3.10 Organoids as a Tool to Study Uterine Fibroid Health Disparity

**FIGURE 9:**
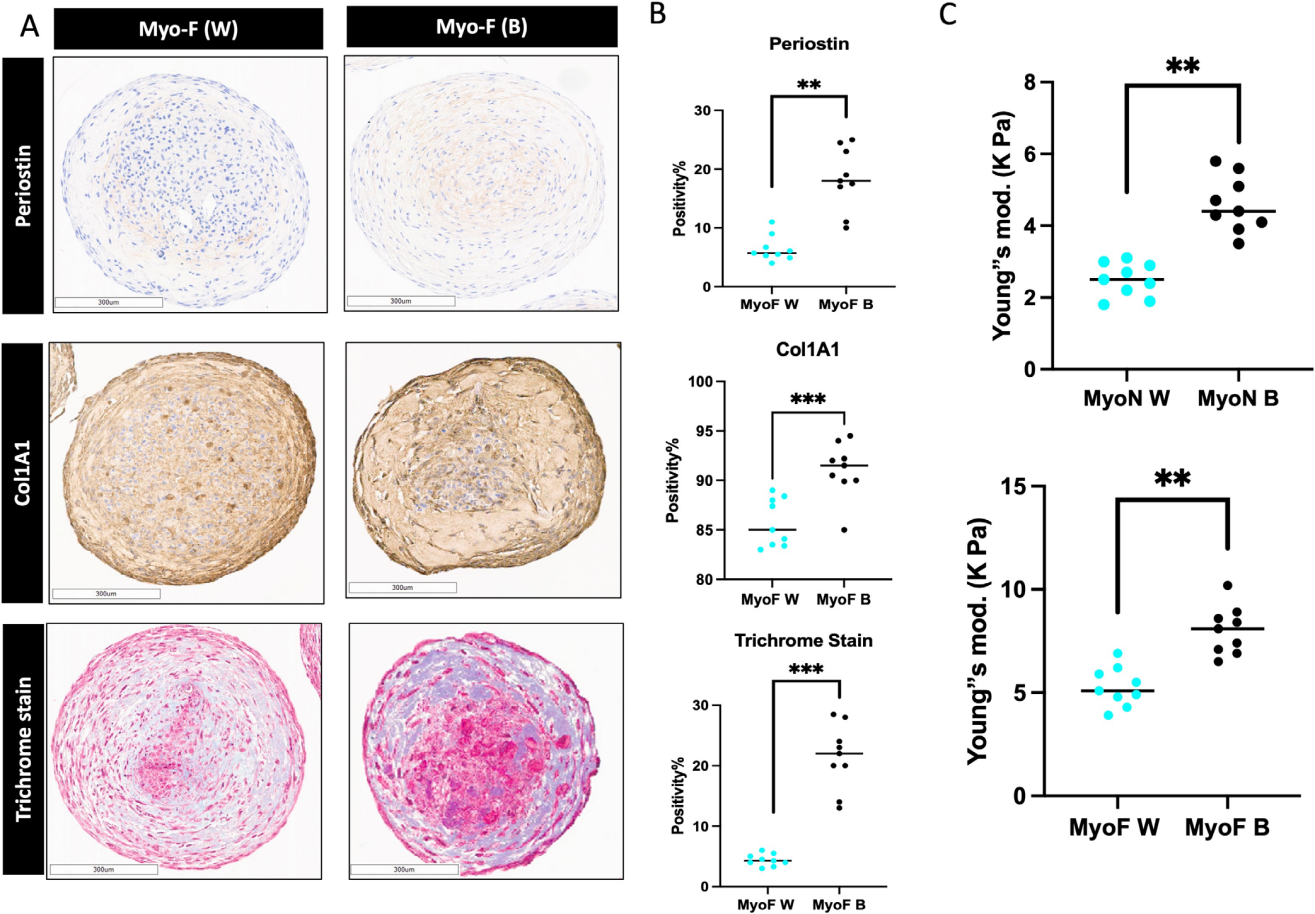
Differences in uterine fibroid health between 3D SCs organoids derived from black and white patients. (A) Immunohistochemical staining showing ECM accumulation in 3D MyoF‐derived organoids from black‐versus white‐type plants expressing periostin (top), COL1A1 and Masson's trichrome staining (bottom). Scale bar = 300 μm. (B) The bar graph is a quantitative representation of IHC staining for Periostin (top) and COL1A1 and Masson's trichrome staining (bottom) of 3D SCs‐derived MyoF organoids derived from black and white patients. The slides were scanned and analysed using the Aperio ImageScope colocalization algorithm Pathology Slide Viewing Software. (C) Young's modulus tested with a single indentation protocol using a Piuma nanoindenter (Optics11, Amsterdam, Netherlands) as an indicator of the stiffness of 3D SCs Myo‐N (top) and MyoF (bottom) derived from black versus white patients. *, **, and *** indicate statistical significance according to Student's *t* test (*p* < 0.05, *p* < 0.01, and *p* < 0.001); ns: Nonsignificant. Each biological replicate is shown as a *circle* and represents the average of three technical replicate measurements.

3D organoids have emerged as powerful models for understanding health disparities. To explore these disparities, we analysed ECM accumulation in 3D MyoF‐derived organoids from self‐identified black and white women using IHC staining. Subjects' characteristics illustrated in Table S3. The results revealed that MyoF derived from black patients exhibited significantly greater percentages of Periostin (18%), COL1A1 (7%), and Masson's trichrome staining (17%) than did MyoF derived from white patients (*p* < 0.01, *p* < 0.01, and *p* < 0.001, respectively) (Figure 9A,B). Theoretically, elevated ECM accumulation could result in stiffer tissue. To corroborate this hypothesis, we assessed the Young's modulus using a single indentation protocol with a Piuma nanoindenter (Optics11, Amsterdam, Netherlands) on 3D SCs MyoN and MyoF derived from black and white patients. Intriguingly, both MyoF B and MyoN B displayed a significantly greater Young's modulus than did MyoF W and MyoN W (*p* < 0.05 and *p* < 0.01, respectively) (Figure 9C). Although there was no significant difference in DNMT activity between MyoN from black and white patients, MyoF B exhibited twofold greater DNMT activity than MyoF W (*p* < 0.05) (Figure S2). As a conclusion, organoids derived from black subjects show higher ECM content and higher stiffness relative to organoids derived from white subjects.

### 3.11 Organoids as a Tool for Drug Screening

**FIGURE 10:**
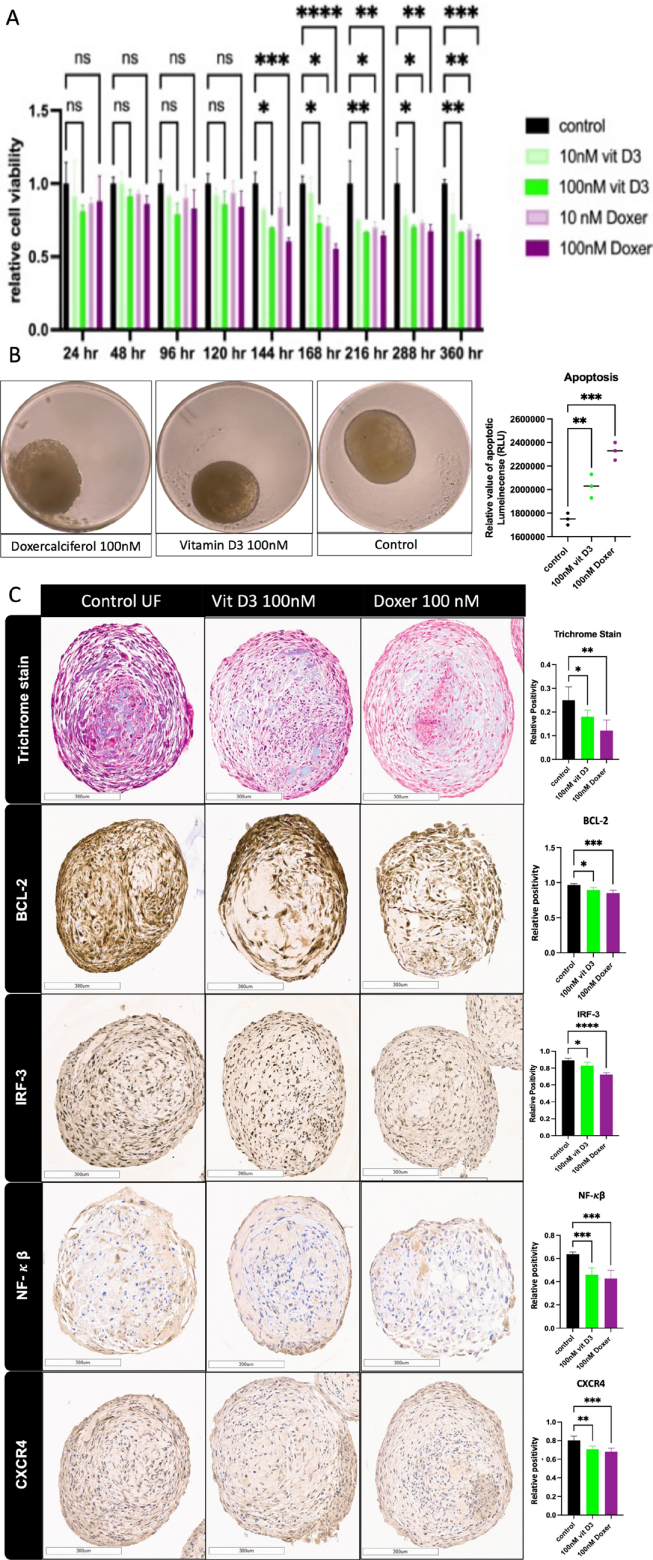
Screening of the antifibrotic effect of Vitamin D3 and Doxercalciferol on UF organoids. (A) Relative viability of UF organoids after exposure to 10 or 100 nM vitamin D3 or Doxercalciferol for up to 15 days. (B) Relative apoptotic luminescence of UF organoids after exposure to 100 nM vitamin D3 or Dox for 15 days. (C) Immunohistochemical staining and the bar graph of quantitative representation of the IHC of 3D UF‐derived organoids treated with 100 nM Vitamin D3 or Doxercalciferol for 15 days; Masson's trichrome staining (top); BCL‐2, IRF‐3, NF‐κβ and CXCR4 (bottom) are shown. Scale bar = 300 μm. The slides were scanned and analysed using the Aperio ImageScope colocalization algorithm Pathology Slide Viewing Software. *, **, and *** indicate statistical significance according to Student's *t* test (*p* < 0.05, *p* < 0.01, and *p* < 0.001); ns: nonsignificant. The bar graphs or circles represent the means ± SEMs of five technical replicate measurements of three independent biological replicates from three different patients.

3D SCs‐derived organoids were used as robust models for screening the antifibrotic effects of different natural and synthetic compounds. UF‐derived 3D organoids were treated with 10 nM or 100 nM vitamin D3 or vitamin D receptor (VDR) agonist Doxercalciferol for up to 15 days, and a significant decrease in cell viability was observed on day 6 (144 h) for the groups treated with 100 nM Doxercalciferol and vitamin D3 and on day 7 (168 h) for the group treated with 10 nM Doxercalciferol (Figure 10A). By measuring the relative number of apoptotic luminescence units, we detected a significant increase in apoptosis in both groups treated with 100 nM Doxercalciferol and vitamin D3 (*p* < 0.001 and 0.01, respectively) (Figure 10B). Masson's trichrome staining, a marker of fibrosis, showed a significant reduction in both groups treated with 100 nM Doxercalciferol and vitamin D3 (*p* < 0.01, 0.05). The groups treated with 100 nM vitamin D3 showed significant decreases in BCL‐2, interferon regulatory factor‐3 (IRF‐3), Nuclear factor kappa B (NF‐κβ) and C‐X‐C motif chemokine receptor 4 (CXCR4) (*p* < 0.05, 0.05, 0.001, 0.01, respectively). Furthermore, BCL‐2, IRF‐3, NF‐κβ and CXCR4 expression significantly decreased in the group treated with 100 nM Doxercalciferol (*p* < 0.001). These findings strongly suggest that these drugs exert their antifibrotic effects through the inflammatory pathway (Figure 10C).

**FIGURE 11:**
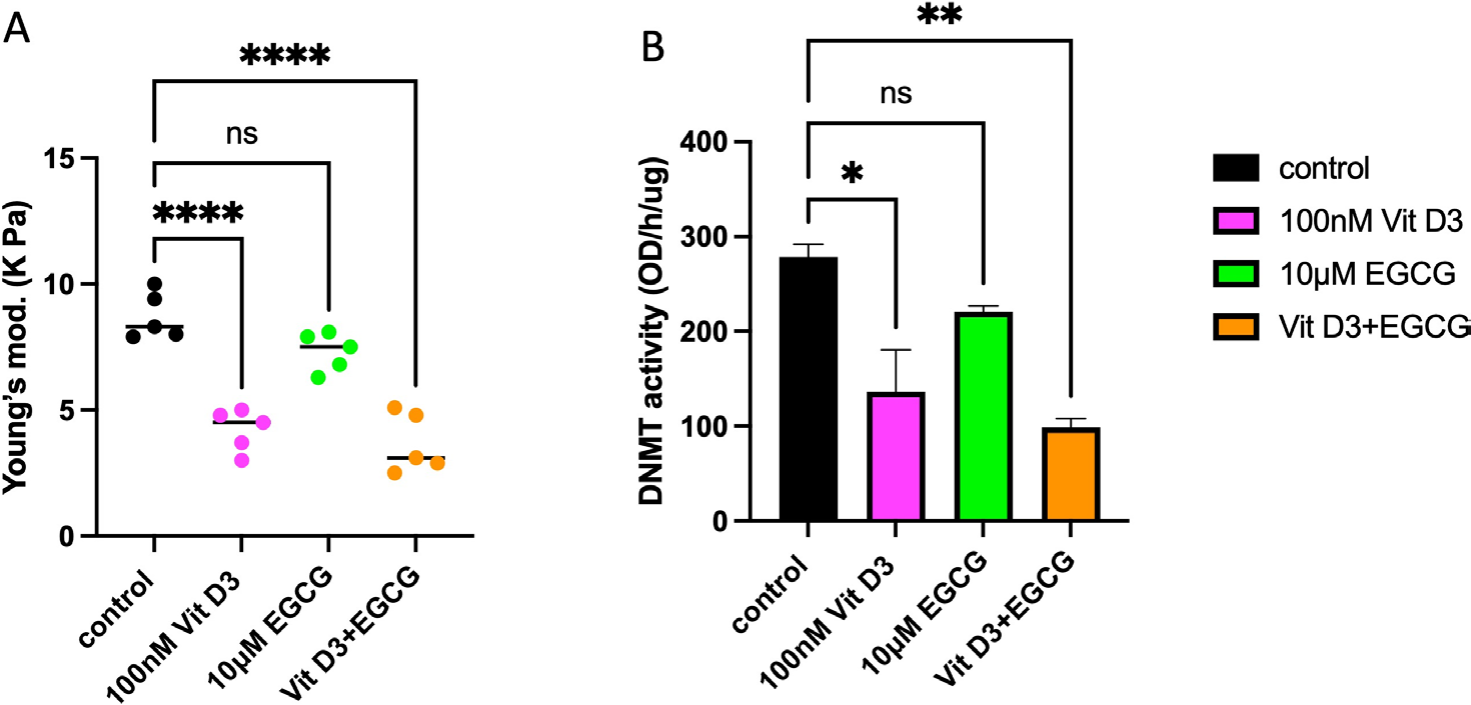
Screening of the antifibrotic effect of 100 nM vitamin D3, 10 μM EGCG and their combination on MyoF 3D organoids. (A) Young's modulus tested with a single indentation protocol using a Piuma nanoindenter (Optics11, Amsterdam, Netherlands) as an indicator of the stiffness. (B) DNMT activity of MyoF 3D organoids after treatment with 100 nM vitamin D3, 10 μM EGCG or their combination. *, **, and *** indicate statistical significance according to two‐way ANOVA with Tukey's post hoc test (*p* < 0.05, *p* < 0.01, and *p* < 0.001); ns: Nonsignificant. The bar graphs or circles represent the means ± SEMs of five technical replicate measurements of three independent biological replicates from three different patients.

Additionally, treatment of MyoF 3D organoids (those considered at risk) with 100 nM vitamin D3, 10 μM EGCG, or their combination significantly decreased the elastic Young's modulus (by one‐fold) in both the vitamin D3‐treated group and the combination group relative to the control group (*p* < 0.001) (Figure 11A). These findings highlight the potential role of the combination of vitamin D3 and EGCG in modulating mechanotransduction as a contributing factor to the uterine fibroid formation process. Additionally, the same pattern was observed for DNMT activity (*p* < 0.05, 0.01) (Figure 11B). This highlights epigenetic activity as a strategic pathway for studying drug mechanisms of action. As a conclusion of this experiment, Doxercalciferol, vitamin D3, and EGCG show promising antifibrotic effects, and further investigation is required to understand their mechanism of action.

### 3.12 Organoids as a Tool to Study the Pro‐Fibroid Effects of Environmental Phthalates

**FIGURE 12:**
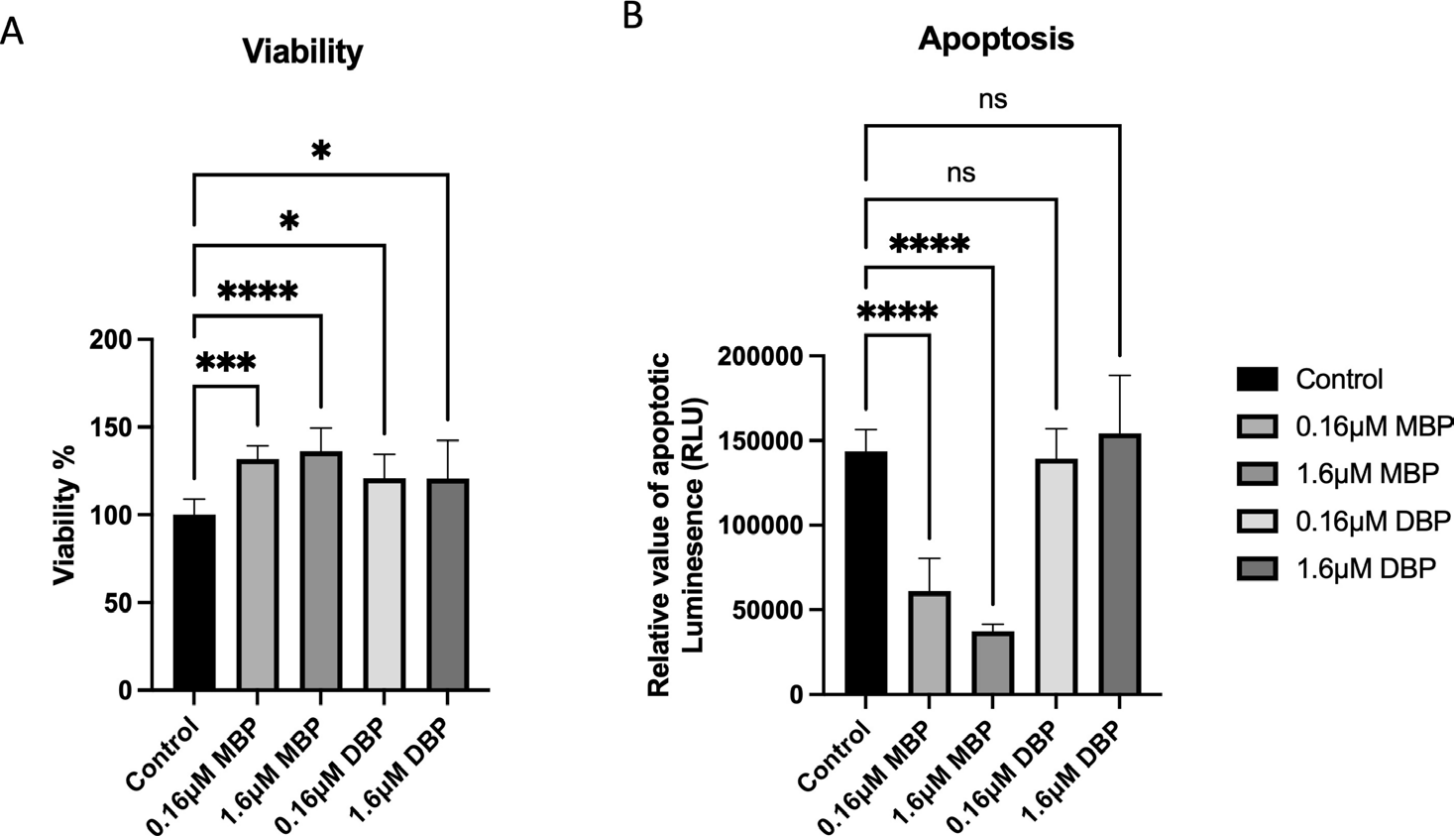
Effect of environmental phthalate on the relative viability of normal myometrial MyoN organoids. (A) Relative viability, (B) relative apoptotic luminescence of MyoN organoids exposed to 0.16 or 1.6 μM MBP or DBP. *, **, and *** indicate statistical significance according to two‐way ANOVA with Tukey's post hoc test (*p* < 0.05, *p* < 0.01, and *p* < 0.001); ns: Nonsignificant. The bar graphs represent the mean ± SEM of five technical replicate measurements of three independent biological replicates from three different patients.

MyoN organoids exposed to 0.16 and 1.6 μM MBP showed significant increases in viability and significant decreases in apoptosis (*p* < 0.05, 0.01). On the other hand, DBP (the parent phthalate) had a less significant effect on viability and a nonsignificant effect on apoptosis. This may be because the parent compound requires a metabolising enzyme that is not available under in vitro conditions (Figure 12A,B).

## 4 Discussion

Uterine fibroids (UFs) stand out as a significant challenge in women's health, affecting 20%–40% of women in their reproductive years for which new therapeutic interventions are needed [36, 37]. Over several decades, collective research efforts have progressively unravelled their multifaceted nature. However, the underlying mechanisms involved in the transformation of myometrial cells into UFs remain unclear. UFs are intricately intertwined with hormonal balance, particularly oestrogen and progesterone, leading to the excessive production of ECM [38, 39, 40]. Diverse factors, including SCs, growth factors, genetic and epigenetic modulators, ovarian steroid hormones, cytokines, chemokines, and ECM components, collaborate and potentially tilt the balance towards UF development and growth [41, 42, 43, 44].

UFs frequently result in HMB throughout a woman's life. Dysfunctional uterine bleeding can be attributed to hormonal imbalances even when the exact cause is unknown [45]. However, emerging evidence indicates that chronic inflammation in the myometrium plays a pivotal role, leading to the secretion of harmful inflammatory substances and thereby contributing to increased bleeding in the endometrium [46]. To better describe this condition, we propose the term “CAM = chronic aseptic myometritis”. This inflammatory milieu, termed CAM, appears to be the harbinger of UF development, signalling its ominous presence even before UFs debut on an ultrasound. Regrettably, CAM is often overlooked in current evaluations, leading to significant implications for understanding HMB, pelvic pain, and preterm laboor in black women, underscoring the importance of screening and prevention. Despite this knowledge, progress in primary and secondary prevention of UFs is hindered by the limited understanding of risk factors contributing to their development.

Historically, the management of UFs has relied heavily on surgical intervention. While effective, hysterectomies are associated with a considerable cost burden and have sweeping implications for public health and affected individuals. Recognising this, research is shifting its focus toward more precise and less invasive solutions. Central to this is the understanding of UF pathophysiology, which mandates models that mirror the in vivo behaviour of these tumours. Here, we present our endeavour to bridge this gap with the introduction of an organoid culture system derived from human SCs from both normal and UF‐afflicted patients. Our team introduced the first UF organoids in 2022 [13], and by comparing our organoids with the previously cultured UFs spheroids [47, 48], our organoids cultured from SCs can be differentiated into two different types of cells while the spheroids cultured from primary cells or immortalised cells can notbe differentiated.

The concept of cellular plasticity has been a focal point in understanding myometrial behaviour. Interestingly, specific cell subsets within the myometrium exhibit stem cell characteristics, both in murine and human models [49, 50, 51, 52, 53]. Genetic irregularities, particularly those targeting the mediator complex subunit 12 (MED12), appear pivotal in UF development, with these mutations conspicuously present only in fibroid SCs [54]. Environmental triggers and the hormonal milieu may further alter the genetic or epigenetic landscape of MMSCs, ultimately paving the way for proliferative, tumour‐initiating cells distinct from their progenitors [55]. Leveraging this insight, we proposed that a 3D organoid model originating from MMSCs could significantly enhance our understanding of UF pathogenesis.

Increasing evidence indicates that putative myometrial stem/progenitor cells may contribute to both physiologic and pathologic processes underlying the formation of UFs; hence, identification of the specific surface markers characterising these cells remains critically important. Previous studies have established that several individual and combined stem/progenitor cell markers, including Oct4 [56], CD34/CD49f/b [57], CD44/Stro1 [15], and CD140b/CD146+ or SUSD2 [58], can be used to identify and characterise cells from the myometrium and UFs. Previously published data also suggest that CD34+/CD49+/+ [59] and/or Stro11+/CD44+/+ [15] cells are comparable and likely to represent the same population of SCs, although they are isolated using different surface markers [60]. As mentioned in the Materials and Methods section and our team's previous publication [15], we selected a subpopulation of myometrial SCs characterised by Stro1/CD44 surface markers due to their demonstrated significance in the initiation and progression of UFs. These markers are associated with key stem cell properties such as self‐renewal and differentiation, which are critical for understanding the cellular dynamics that contribute to fibroid development and growth. Diving deep into the molecular complexities of UFs, one is struck by the complexity. UFs harbour more genetic aberrations than their normal counterparts, a discrepancy that might arise from diminished DNA repair [61].

Clonality has always been a cornerstone in understanding tumours. In the context of UFs, while they originate from a single smooth muscle cell clone, as they expand, they differentiate into fibroblast and smooth muscle cell subpopulations [62]. The role of the ECM in UF growth cannot be overstated. It not only forms the structural framework but also acts as a reservoir for a myriad of growth modulators, including cytokines, growth factors, and chemokines [38, 40, 63, 64, 65, 66, 67, 68]. Our findings align with this narrative, showing increased expression of key ECM components in derived organoids, indicating their predisposition toward UFs. Our study revealed that CD44/Stro1++ SCs isolated from human MyoN (normal myometrium), MyoF (at risk) and UF (fibroid) biopsies grown in three‐dimensional culture under normoxic conditions expressed a mixed population of the stem cell markers Stro‐1 and CD44, alpha‐smooth muscle actin, the stromal cell marker vimentin, and the ECM markers COL1A1, COL3A1, and FN.

The growth of UFs is characterised by slow proliferation and is associated with increased production and concurrent deposition of ECM proteins, usually in a steroid hormone‐dependent manner [40]. The (ECM) is involved in most tumour growth and consists mainly of glycoproteins, collagens, and peptidoglycans [63, 64]. Some of the abundant and upregulated proteins in fibroids are COL1A1, FN1, and periostin [38, 63, 65]. The ECM represents the pathological microenvironment, which serves as a reservoir for growth factors, cytokines, chemokines, and inflammatory response mediators [66, 67, 68]. The ECM imparts abnormal stiffness to the UF, resulting in increased mechanical stress and making it inaccessible to therapeutic agents [69, 70]. Previous studies defined the proteome of UFs and established that increased periostin production is a hallmark of UFs regardless of the *MED12* mutation status [63]. Consistent with established findings, our results demonstrate that, compared with MyoN organoids, CD44/Stro1++ stem cell‐derived UF organoids and MyoF at risk organoids express elevated levels of periostin, COL3A1, COL1A1, and FN. This supports the validity of our organoid system as a reliable model for reflecting the ECM dynamics known to contribute to the formation of UFs.

In the current study, UF organoids exhibit a significant 3‐fold increase in TGFβ3 expression relative to MyoN. This finding is consistent with the findings of previous studies that show that the TGF‐β expression in the smooth muscle of the uterus, which is in direct contact with the fibroid (MyoF), is significantly increased [71]. Additionally, the expression of TGF‐β in UF tissue as compared to the control group (normal smooth muscle) is almost twice as high [72]. Interestingly, the TGF‐β3 isoform occurs in UF tissue at concentrations almost five times higher than in the healthy myometrium [73].

In the present study, comparisons of MyoF‐MyoN, UF‐MyoF, and UF‐MyoN samples also revealed significant differences in gene expression patterns, especially in the ECM. Only a small number of genes showed consistent changes in the same direction, and the majority exhibited opposite changes. This suggests that the transition from normal to at risk myometrium to complete conversion to UF involves different gene changes/signalling pathways. Nevertheless, the pathway analysis of MyoF‐MyoN, UF‐MyoF, and UF‐MyoN further emphasised the involvement of ECM‐related pathways. We then focused on evaluating ECM components that excessively accumulate in UFs, such as COL1A1, COL3A1, and FN, to explore whether their enhanced production can also be observed in the at risk myometrium compared to the normal myometrium. We noted increased levels of COL1A1, COL3A1, and FN proteins in the UF and MyoF organoids compared with those in the MyoN organoids. These results suggest that ECM accumulation is involved in the transition of normal myometrium to at risk myometrium, which puts these patients at risk for developing fibroids and other risk factors. This finding confirmed the findings of a previous studies by Bariani et al. and Bateman et al. [74, 75]. Overall, our observations indicate that the racial disparities observed in UF disease may be attributed, at least in part, to exacerbated production of ECM in the myometrium of black women, even before the appearance of the tumours. This potential molecular observation implies that enhanced ECM accumulation increases the stiffness of the tissue, which consequently triggers mechanotransduction signals, resulting in the production of more ECM components, a phenomenon called dynamic reciprocity [76]. Importantly, dysregulated mechanotransduction has been shown to be related to enhanced inflammation [77, 78], and inflammation is connected with fibrosis [79].

DNA methylation appears to be established by a complex interplay of DNA methyltransferases. DNMT1 plays a role in maintaining DNA methylation patterns during DNA replication [80, 81]. Our study revealed an increase in overall DNMT activity in UFs and MyoF relative to that in MyoN. The expression of DNMT1, DNMT3A, and DNMT3B in human UFs has been found to differ from that in the adjacent myometrium [82, 83]. In samples from African‐American, Caucasian, and Hispanic women, the mRNA expression levels of DNMT3a and DNMT3b were lower in UFs than in the myometrium. In contrast, the expression of DNMT1 in UFs was greater than that in the myometrium [84]. On the other hand, a previous study reported that, in Japanese women, DNMT1 and DNMT3a mRNA expression levels were greater in UFs than in the myometrium. In contrast, there was no significant difference in DNMT3b mRNA expression between UFs and the myometrium [18]. The increased DNMT1 expression that was found in both studies may reflect an elevated proliferative activity of uterine fibroid cells because DNMT1 is responsible for copying methylation patterns following DNA synthesis [80, 81]. However, the two studies differed in their findings on the relative expression of DNMT3a and DNMT3b. The reason for the discrepancies is unclear but may be due to race‐dependent differences.

The stem cell‐derived organoids self‐organise to mimic the in vivo structural organisation, as smooth muscle actin cells organise to the exterior of the organoid with few labelled cellular structures within the interior. Within 7 days, these SC‐derived organoids recapitulate many of the physiologically relevant properties and features of the in vivo tissue model, thus revealing new possibilities for investigating the BP involved in disease modelling and testing patient‐specific drugs. Epidemiological, clinical, and experimental evidence supports the pivotal role of ovarian steroid hormones in the growth and pathogenesis of UFs. The effects of (E2) and (P4) are interrelated and involve the mediation of receptors, transcription factors, kinase proteins, growth factors, and numerous autocrine and paracrine factors [84]. In the present study, MMSC organoids responded to ovarian hormones and increased oestrogen receptor expression in the treated groups. Previous studies have suggested that E2 predominantly increases tissue sensitivity to progesterone by increasing the availability of progesterone receptors and that P4 is required for the complete development and proliferation of UF cells. However, there are conflicting results available about the role of progesterone in UF development, as it can be either stimulatory or inhibitory [85]. Additionally, progesterone increased proliferation and inhibited apoptosis in 3D myometrial organoids. This finding is different from that of a previous study on primary cultures of uterine leiomyoma cells [86].

Using organoids as a tool for drug screening revealed the promising effect of the combination of EGCG and vitamin D3 on decreasing the stiffness of myometrium at risk (MyoF) organoids. This finding emphasises the synergistic potential of this combination in the treatment and prevention of UFs, whereas EGCG alone did not show a statistically significant reduction in stiffness. A recent study showed that EGCG reduced the expression of ECM proteins and reduced the expression of downstream and upstream mediators of fibrosis in uterine fibroid cells [87]. Additionally, a previous pilot clinical trial suggested that supplementation with EGCG and Vit D3 reduced the size of UFs and abnormal heavy bleeding. Therefore, this novel combination could be an alternative approach to “wait and see” for UF reduction and associated symptoms [88].

On the other hand, the current study revealed that vitamin D3 and Doxercalciferol decrease proliferation and increase apoptosis in uterine fibroid organoids through the inhibition of several inflammatory markers. This finding suggested that the administration of vit D3 and its analogues may reduce the size of leiomyomas. It seems that their administration may be an effective way to treat leiomyoma. A previous clinical trial suggested the same for vitamin D3 [89].

Exposure to some phthalate biomarkers was positively associated with uterine volume, which further supports the hypothesis that phthalate exposure may be associated with fibroid outcomes [90]. The current study used organoids as tools to test the effect of exposure to di‐butyl phthalate and its metabolite mono‐butyl phthalate on normal myometrial organoids.

Black individuals are diagnosed with fibroids approximately three times as frequently as white people, develop fibroids earlier in life, and tend to experience larger and more numerous fibroids that cause more severe symptoms [91]. Our work used organoids as a tool to better understand the health disparity of UFs. The 3D at risk MyoF‐derived organoids from black patients had more ECM and were stiffer than the 3D at risk MyoF‐derived organoids from white patients. Further in‐depth investigations using 3D organoids may help to elucidate this complicated pathophysiology.

The advantage of our technique includes: contribution to reduction in animal testing, it can be used for functional assays, cytopathology, molecular analysis, and drug testing within a short time frame. Our organoids are homogenous, regular, circular in size and shape. The organoids produced by our research team mimic the structural and functional properties of actual UFs. Although these organoids do not form blood vessels or glandular structures but they respond to hormones like oestrogen. It is crucial to conduct future targeted experiments to address more specific characterisation. On the other hand, the main limitation is that further investigation is required to establish an organoids co‐culture system to include more types of cells like immune cells.

In summary, our research sheds light on the potential impact of the ECM on functional and cellular responses in SCs derived organoids. These insights contribute to a deeper understanding of the molecular mechanisms involved in the development of UFs, paving the way for exploring targeted therapeutic interventions. Moreover, further research investigating the long‐term effects of the ECM and its implications for fibroid development in vivo is important. By gaining a comprehensive understanding of the relationship between the ECM and UFs' pathogenesis, we may identify novel prevention‐based therapies and improve clinical outcomes for affected individuals. By revealing the secrets of the ECM and its pivotal role in UF pathogenesis, we hope to herald a new era in therapeutic innovation and preventive strategies, diminishing the shadows cast by UFs on countless lives.

In conclusion, our organoid model has unlocked a promising vista in UF research, faithfully recapitulating the ECM dynamics observed in UFs. Serving as a powerful preclinical tool, this system holds promise for demystifying the molecular underpinnings of UFs, potentially steering us toward innovative therapeutic avenues.

## Author Contributions

M.M.O. made the major contribution to the acquisition, analysis, and interpretation of the data, and drafted the manuscript. Conceptualization, A.A.‐H., M.M.O., M.A.; Q.Y., W.E.T.; Methodology, M.M.O., T.B.; Software and formal analysis, M.M.O.; Investigation, resources, and data curation, M.M.O., S.V.; Writing original draft preparation, M.M.O., S.A.; Review and editing, M.M.O., A.A.‐H., S.V., F.L.A., M.V.B., S.A., M.A., Q.Y.; Visualisation, supervision, project administration, and funding acquisition, A.A.‐H., M.A. All authors have read and agreed to the published version of the manuscript.

## Consent

Informed consent was obtained from all subjects involved in the study according to the tissue bank of the University of Chicago IRB#20–1414. Written informed consent was obtained from the subjects to publish this paper. The manuscript was reviewed and approved by all the authors.

## Conflicts of Interest

The authors declare no conflicts of interest. The National Institutes of Health provided Dr. Ayman Al‐Hendy reports. Ayman Al‐Hendy reported a relationship with Myovant Sciences Ltd and Pfizer, including consulting or advisory relationships. Additionally, Dr. Ayman Al‐Hendy is the founder of the INOFFA Company.

## Supporting information


Data S1.

## Data Availability

The raw data were generated at the University of Chicago. Derived data supporting the findings of this study are available from the corresponding author upon request. The data are not publicly available because they contain information that could compromise participants' ethical approval and consent to participate.
