# Supplementary material for: Utilising Human Myometrial and Uterine Fibroid Stem Cell‐Derived Three Dimentional Organoids as a Robust Model System for Understanding the Pathophysiology of Uterine Fibroids
Source: Cell Prolif. 2025 Mar 20;58(9):e70025. doi: 10.1111/cpr.70025 (PMC12414642; doi:10.1111/cpr.70025)
Supplement: Supplementary file 1 — Data S1. [file CPR-58-e70025-s001.docx]

**Table S1:** List of genes and primers used for qRT-PCR.

| **Symbol** | **Forward primer sequence (5’-3’)** | **Reverse primer sequence (5’-3’)** |
| --- | --- | --- |
| GAPDH | GTCTCCTCTGACTTCAACAGCG | ACCACCCTGTTGCTGTAGCCAA |
| Cyclin D | GTTGCAAAGTCCTGGAGCCT | CACAGGAGCTGGTGTTCCAT |
| β-Catenin | CACCCGCAGAGTGCTGAAGGTG | GATTCCTGAGAGTCCAAAGACAG |
| TGFβ3 | CACACAGTCCGCTTCTTC | AGAAGAGGGTGGAAGCC |
| Fibronectin | ACAACACCGAGGTGACTGAGAC | GGACACAACGATGCTTCCTGAG |
| Col1A1 | GATTCCCTGGACCTAAAGGTGC | AGCCTCTCCATCTTTGCCAGCA |
| Col3A1 | TGGTCTGCAAGGAATGCCTGG | TCTTTCCCTGGGACACCATCA |

**Table S 2:** List of primary antibodies used in experiments.

| **Antibodies names** | **Type** | **Using in study** | **Optimized Dilution** | **Source** |
| --- | --- | --- | --- | --- |
| Anti-PCNA antibody [PC10] (ab29) | Primary | IHC | 1:1000 | (Abcam, MA, USA) |
| Anti-Bcl-2 antibody (ab59348) | Primary | IHC | 1:250 | (Abcam, MA, USA) |
| Anti-NF-kB p65 antibody [E379] (ab32536) | Primary | IHC | 1:5000 | (Abcam, MA, USA) |
| Anti-IRF3 antibody [EPR2418Y] (ab68481) | Primary | IHC | 1:500 | (Abcam, MA, USA) |
| Anti-Cyclin D [SP4] (ab16663) | Primary | IHC/IF | 1:100 | (Abcam, MA, USA) |
| Anti-Estrogen Receptor alpha antibody (ab3575) | Primary | IHC | 1 µg/ml | (Abcam, MA, USA) |
| Anti-Periostin antibody [EPR20806] (ab215199) | Primary | IHC | 1:500 | (Abcam, MA, USA) |
| β-Catenin antibody [E247] - ChIP Grade(ab32572) | Primary | IHC/IF | 1:500 | (Abcam, MA, USA) |
| Anti-Fibronectin antibody (ab2413) | Primary | IHC | 1:400 | (Abcam, MA, USA) |
| Col1A1 (E8F4L) XP® Rabbit mAb #72026 | Primary | IHC | 1:400 | Cell signaling technology, USA) |
| Anti-Collagen III antibody (ab7778) | Primary | IHC/IF | 1:500 | (Abcam, MA, USA) |
| Anti-Ahr [RPT] (GTX227700a) | Primary | IF | 1:1000 | ( GeneTex , Taiwan) |
| STRO-1 Monoclonal Antibody (STRO-1), (14-6688-82) eBioscience | Primary | IF | 20 µg/mL | (Invitrogen, USA) |
| [Anti-alpha smooth muscle Actin antibody [1A4] (ab7817)](https://www.abcam.com/products/primary-antibodies/alpha-smooth-muscle-actin-antibody-1a4-ab7817.html) | Primary | IF | 1 µg/ml. | (Abcam, MA, USA) |
| Anti-Vimentin antibody [LN-6] (ab230171) | Primary | IF | 1 µg/ml | (Abcam, MA, USA) |
| Stro-1 Anti-STRO1 antibody (PE) (sc-47733) | Conjugated | Flow | 1:100 | (Santa Cruz biotechnology, USA) |
| CD44 FITC Mouse Anti-Human CD44 (#555478) | Conjugated | Flow | 20 µl | (BD Biosciences, USA) |
| Anti-α-SMA (ab225143) | Conjugated | Flow | 1:5000 | (Abcam, MA, USA) |
| Vimentin antibody anti human, FITC, REAfiniity clone REA409 | Conjugated | Flow | 1:50 | (MiltenyiBiotic, Gernmany) |
| Alexa Fluor 488 goat anti mouse IgG (A11029) | Secondary | IF | 1:1000 | (Life Technologies, CA, USA) |
| Alexa Fluor 594 goat anti rabbit IgG (A11037) | Secondary | IF | 1:1000 | (Life Technologies, CA, USA) |

**Table S3:** Table of subjects’ characteristics from their tissue the stem cells isolated.

| **Subject code** | **phenotype** | **Sample collected** | **age** | **race** | **BMI** | **Stem cell type isolated** |
| --- | --- | --- | --- | --- | --- | --- |
| 514 | control | NORMAL Myometrium | 37 | Black | NA | MyoN |
| 518 | control | NORMAL Myometrium | 29 | White | NA | MyoN |
| UI-36 | control | NORMAL Myometrium | NA | Caucasian | NA | MyoN |
| UI-28 | control | NORMAL Myometrium | NA | African American | NA | MyoN |
|  |  |  |  |  |  |  |
| UI-41 | Fibroids | Myometrium/Fibroid | 53 | Black | 48.26 | UF/MyoF |
| UI-42 | Fibroids | Myometrium/Fibroid | 36 | Black | 30.86 | UF/MyoF |
| UI-18 | Fibroids | Myometrium/Fibroid | 53 | Black | 39.87 | UF/MyoF |
| UI-34 | Fibroids | Myometrium/Fibroid | 36 | Black | 41.03 | UF/MyoF |
| 515 | Fibroids | Myometrium/Fibroid | 45 | White | NA | UF/MyoF |
| 512 | Fibroids | Myometrium/Fibroid | 46 | White | NA | UF/MyoF |

*NA: data not available

**Figure S1:** A) Flow cytometry of dissociated SCs-derived organoids using DAPI-free (life cells) (top) and vimentin/α-SMA (bottom right), Stro-1/CD44 (bottom left). B) The graph shows the relative percentages of live cells and CD44/Stro-1-, Vimentin and α-SMA-expressing cells. All the data are from three independent (n = 3) experiments performed for each individual group. Images were acquired using an Olympus BX41 microscope (Olympus America, Center Valley, PA). The images were analyzed by QuPath software for bioimaging analysis. The bar graphs represent the mean ± SEM of three technical replicate measurements of three independent biological replicates.

**
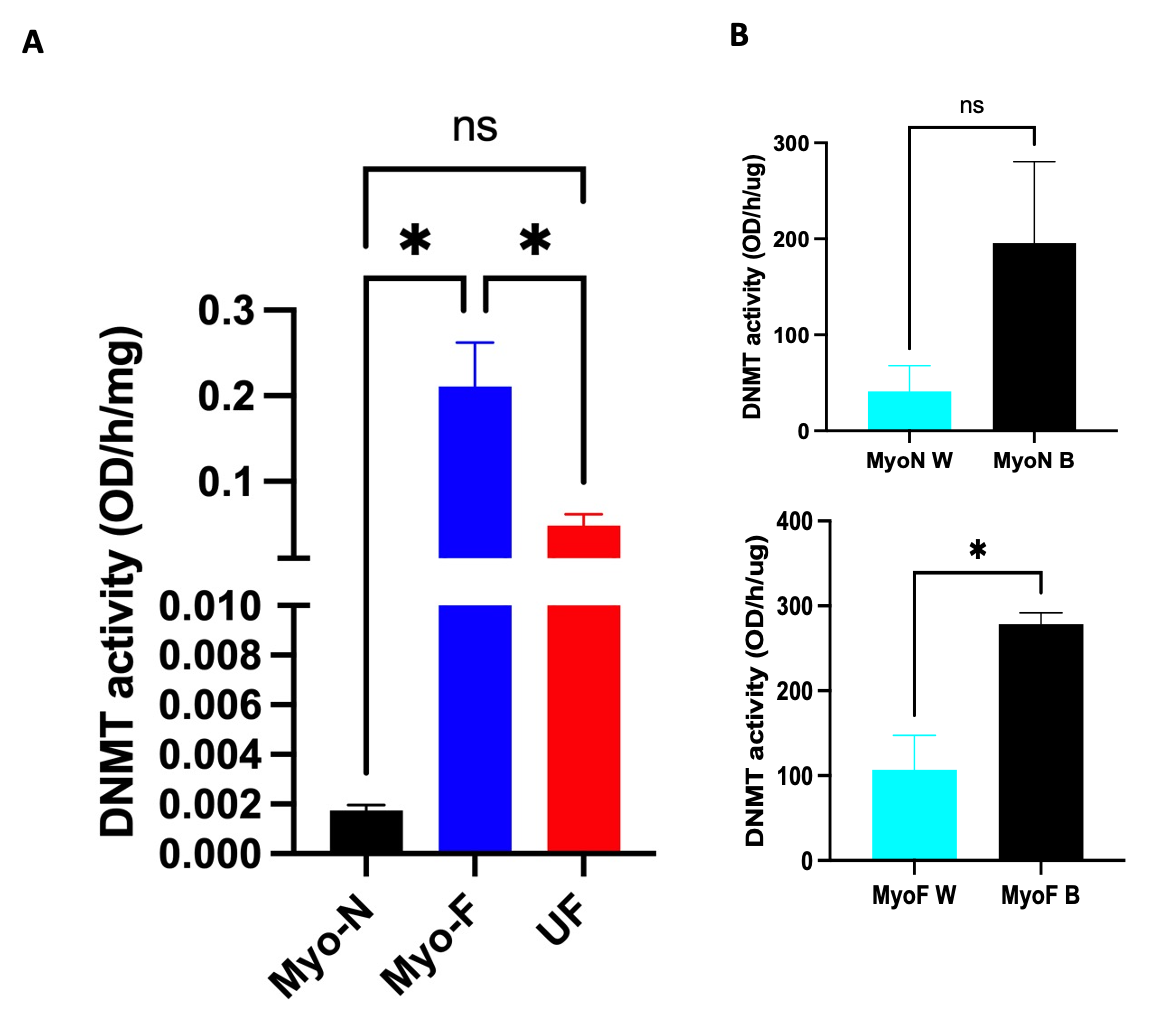
**

**Figure S2:** A) Total DNMT activity of the 3D SCs-derived organoids MyoN, MyoF and UF. . B) DNMT activity of 3D SCs Myo-N (top) and Myo-F (bottom) derived from black versus white patients. *, **, and *** indicate statistical significance according to two-way ANOVA with Tukey's post hoc test and Student’s t test (p < 0.05, p < 0.01, and p < 0.001); ns: nonsignificant. The bar graphs represent the mean ± SEM of three technical replicate measurements of three independent biological replicates from three different patients.

**
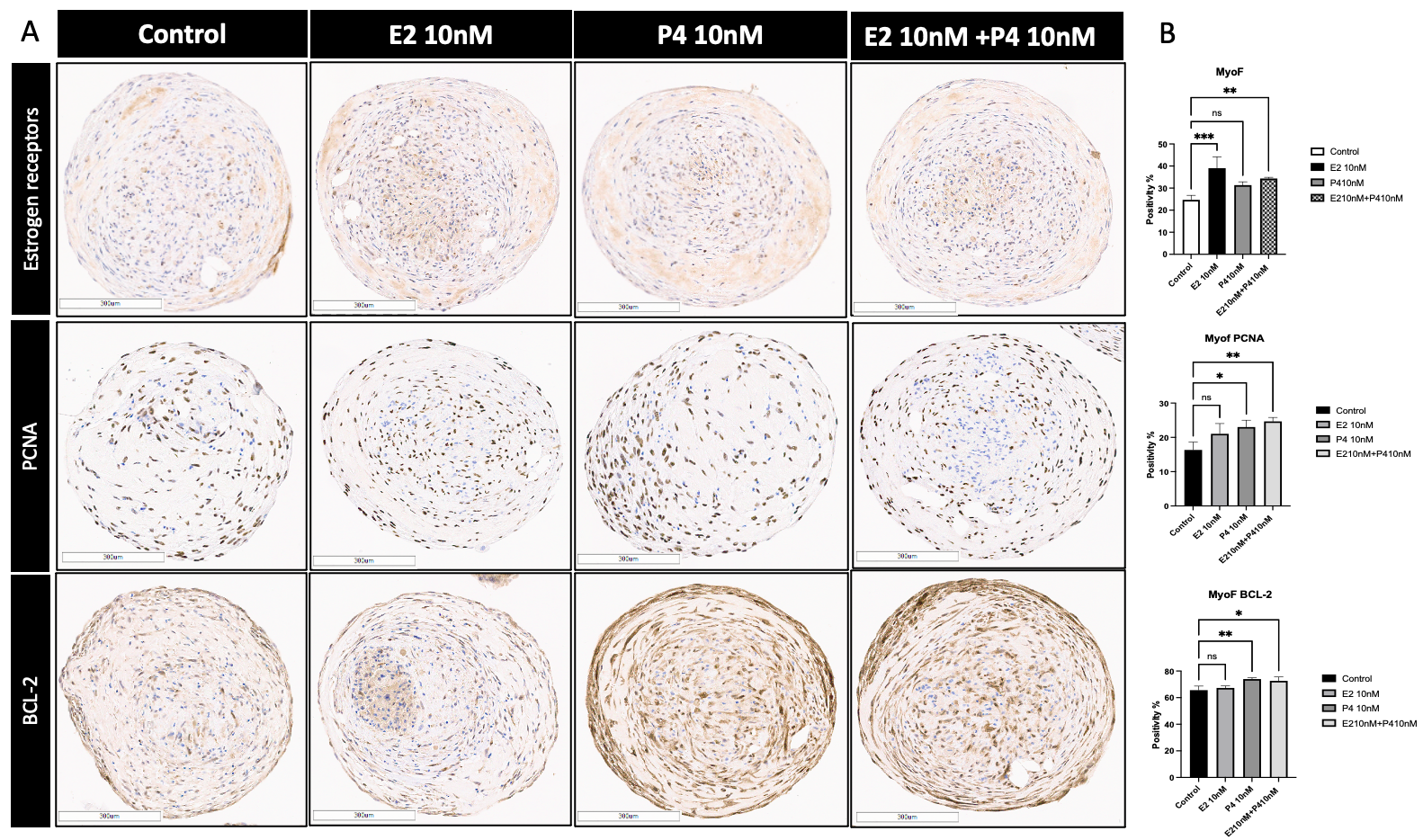
**

**Figure S3:** Response of SCs-derived organoids treated with estradiol (E2, 10 ng/ml), progesterone (P4, 10 ng/ml) or their combinations to ovarian steroid hormones for 48 h. A) Immunohistochemical staining for the estrogen receptor, PCNA and BCL-2 in MyoF SCs-derived organoids. B) The bar graph is a quantitative representation of the IHC staining of the ER antigen, PCNA and BCL-2 on the SCs-derived MyoF organoids in response to E2 and P4. The slides were scanned and analyzed using the Aperio ImageScope colocalization algorithm Pathology Slide Viewing Software. *, **, and *** indicate statistical significance according to two-way ANOVA with Tukey's post hoc test (p < 0.05, p < 0.01, and p < 0.001); ns: nonsignificant. The bar graphs represent the mean ± SEM of five technical replicate measurements of three independent biological replicates from three different patients.
